# Screening and identification of an aflatoxin B_1_-degrading strain from the Qinghai-Tibet Plateau and biodegradation products analysis

**DOI:** 10.3389/fmicb.2024.1367297

**Published:** 2024-05-01

**Authors:** Ying Tang, Xiaojing Liu, Ling Dong, Shengran He

**Affiliations:** College of Pratacultural Science, Gan Su Agricultural University, Lanzhou, China

**Keywords:** aflatoxin B1, *Bacillus amyloliquefaciens* YUAD7, biological detoxification, detoxification mechanism, degradation products

## Abstract

This research aimed to address the issue of aflatoxin B_1_ (AFB_1_) contamination, which posed severe health and economic consequences. This study involved exploring unique species resources in the Qinghai-Tibet Plateau, screening strains capable of degrading AFB_1_. UPLC-Q-Orbitrap HRMS and NMR were employed to examine the degradation process and identify the structure of the degradation products. Results showed that *Bacillus amyloliquefaciens* YUAD7, isolated from yak dung in the Qinghai-Tibet Plateau, removed 91.7% of AFB_1_ from TSB-AFB_1_ medium with an AFB_1_ concentration of 10 μg/mL (72 h, 37°C, pH 6.8) and over 85% of AFB_1_ from real food samples at 10 μg/g (72 h, 37°C), exhibiting strong AFB_1_ degradation activity. *Bacillus amyloliquefaciens* YUAD7’s extracellular secretions played a major role in AFB_1_ degradation mediated and could still degrade AFB_1_ by 43.16% after boiling for 20 min. Moreover, *B. amyloliquefaciens* YUAD7 demonstrated the capability to decompose AFB_1_ through processes such as hydrogenation, enzyme modification, and the elimination of the -CO group, resulting in the formation of smaller non-toxic molecules. Identified products include C_12_H_14_O_4_, C_5_H_12_N_2_O_2_, C_10_H_14_O_2_, C_4_H_12_N_2_O, with a structure consisting of dimethoxyphenyl and enoic acid, dimethyl-amino and ethyl carbamate, polyunsaturated fatty acid, and aminomethyl. The results indicated that *B. amyloliquefaciens* YUAD7 could be a potentially valuable strain for industrial-scale biodegradation of AFB_1_ and providing technical support and new perspectives for research on biodegradation products.

## 1 Introduction

Aflatoxin (AFT) is a highly carcinogenic and teratogenic mycotoxin, primarily produced as a secondary metabolite by *Aspergillus flavus* and *Aspergillus parasiticus fungi* (Maxwell et al., 2021). AFT contamination affected 60–80% of food and feed worldwide, particularly products derived from various types of silage, resulting in annual economic losses reaching trillions (Song et al., 2022). Among the 20 types of aflatoxins identified, aflatoxin B_1_ (AFB_1_) is the most toxic and has been classified as a Group I human carcinogen by the International Agency for Research on Cancer (IARC; Suman, 2021; Zhang et al., 2024). Furthermore, although there have been some physical and chemical techniques for AFB_1_ removal, defects, including inefficient detoxification, nutrient preservation, costliness, and toxic by-products, existed before widespread implementation (Yan et al., 2022; Loi et al., 2023). Consequently, searching for safe and effective large-scale AFB_1_ detoxification strategies has become a focal point of current scientific research.

Microbial degradation is an attractive technology for AFB_1_ cleanup due to its high specificity efficiency and ecological sustainability without compromising food safety. Several beneficial microorganisms had identified for reducing AFB_1_ levels in contaminated media according to the researchers, including *Pseudomonas* spp. (Adebo et al., 2016; Biessy and Filion, 2021), *Rhodococcus* spp. (Risa et al., 2018; Alvarez et al., 2019), *Bacillus* spp. (Xia et al., 2017; Xu et al., 2017; Shu et al., 2018; Wang Y. et al., 2018), *Escherichia coli* (Wang L. et al., 2018), *Pleurotus* spp. (Sharma et al., 2021), and *Aspergillus niger* (Yu et al., 2021). While numerous strain capable of degrading AFB_1_ have been screened, particularly *Bacillus* strain stood out as attractive candidates due to their high degradation efficiency (Yang et al., 2023). For example, Wang Y. et al. (2018) observed that *B. licheniformis* BL010 reduced AFB_1_ levels by 89.1% (120 h, 30°C, AFB_1_ concentration of 0.5 μg/mL). Shu et al. (2018) found that *B. velezensis* DY3108 reduced AFB_1_ levels by 82.0% (within 72 h, 30°C, AFB_1_ concentration of 5 μg/mL). Despite the outstanding degradation performance exhibited by the screened *Bacillus* strains at low AFB_1_ concentrations (not exceeding 5 μg/mL), this is insufficient to meet the requirements for industrially treating high-concentration AFB_1_ contamination. Industrial AFB_1_ pollution concentrations can reach as high as 10 μg/g, and there are still no strains capable of effectively removing AFB_1_ at such elevated concentrations. Previous research indicates that strains screened from extreme environments, particularly the Qinghai-Tibet Plateau, possess stronger temperature resistance, stress tolerance, and unique functionalities than strains selected under other conditions (Bai et al., 2021). The research enriched and domesticated strains from extreme environments aim to address large-scale and high-concentration AFB_1_ pollution in industrial settings, which provides a new resource for the industrial-scale removal of high-concentration AFB_1_.

Although biodegradable products are often considered safe, it is essential to assess their safety, as the degraded products may still be hazardous. In recent years, some technological approaches have been used to study the biodegradation products of AFB_1_. It was determined the composition and chemical formula of AFB_1_ degradation products by HPLC-Q-TOF-MS in tea-derived *Aspergillus niger* RAF106 (Fang et al., 2020), and investigated the degradation products of AFB_1_ by two Bacillus Strains using LC-Triple-TOF-MS (Wang et al., 2022). As mentioned above, mass spectrometry was used to detect the degradation products of AFB_1_, which could only determine the chemical composition and formula of the products. The structural information of the products could only be inferred based on software calculations by mass spectrometry. Additionally, the interdisciplinary research applied NMR to determine the composition and structures of compounds in complex samples (Zhu et al., 2019; Reif et al., 2021). This study utilizes NMR, which has high sensitivity and resolution, and can generate two-dimensional and multidimensional spectra, making it a powerful technique for determining complex compound structures. It is essential to ensure the safety and effectiveness of biodegradation pathways by identifying the composition and structures of biodegradation products of AFB_1_. Defining the degradation pathway will contribute to selectively screening for microbial strains that effectively degrade AFB_1_ while minimizing potential hazards.

The research focused on screening strains capable of efficiently degrading AFB_1_ from the Qinghai-Tibet Plateau’s extreme environment, which was easily found in ensiled forage and animal manure. We used UPLC-Q-Orbitrap HRMS to determine the chemical composition of the products and applied NMR to elucidate the structure of degradation products, thereby predicting the degradation pathway. This experimental screening of AFB_1_-degrading strains from the Qinghai-Tibet Plateau enriches the biological resources available for AFB_1_ degradation and introduced novel insights into the study of the composition, structure, and degradation pathways of these products.

## 2 Materials and methods

### 2.1 Isolation and purification of the target strain

From July to October 2022, samples (Silage corn, Silage alfalfa, and animal feces) were collected from the Qinghai-Tibet Plateau region, including Diebu County, Xiahe County, Gaotai County and Sunan County, Tianzhu Tibetan Autonomous County in Gansu Province. Xining City, Haixi Mongolian and Tibetan Autonomous Prefecture in Qinghai Province. Each 25 g sample was thoroughly mixed with 225 mL of sterile physiological saline solution. Subsequently, a gradient dilution was performed to achieve dilution levels of 10^−3^~10^−7^. All dilutions were evenly spread onto a culture medium with coumarin as the sole carbon source (CM medium; L-1distilled water): 10 g coumarin, 0.25 g KH_2_PO_4_, 1 g NH_4_NO_3_, 1 g CaCl_2_, 0.25 g MgSO_4_·7H_2_O, 1 mg FeSO_4_, and 15 g agar (Rao et al., 2017). The cultures were then incubated at 37°C for 72 h. Colonies displaying growth on the CM plates were chosen for the degradation and rescreening experiments of AFB_1_.

### 2.2 Determination of AFB_1_ degradation in liquid culture medium

The preparation method for TSB-AFB_1_ liquid medium was as follows: Take 1 mL of AFB_1_ standard solutions (Sigma-Aldrich, St. Louis, MO, United States) with concentrations of 200, 400, 600, 800, 1,000 μg/mL, and add them to 100 mL of TSB liquid medium, respectively. The final AFB_1_ concentrations in TSB-AFB_1_ liquid medium were 2, 4, 6, 8, 10 μg/mL, with the pH adjusted to 6.8. Following a modified method based on Chen et al. (2022), a 10 mL bacterial suspension with a concentration of 10^8^ CFU/mL was inoculated into 100 mL TSB-AFB_1_ liquid medium (with AFB_1_ concentrations of 2, 4, 6, 8, 10 μg/mL), and the cultures were shaken culture (37°C, 180 rpm, 72 h). A sterile TSB-AFB_1_ was used as a control. The second screening of initial strains was based on their ability to degrade AFB_1_ in TSB-AFB_1_ medium with a concentration of 10 μg/mL AFB_1_. Evaluated the degradation ability of the finally selected strains toward varying concentrations of AFB_1_ using TSB-AFB_1_ medium with different concentrations (2, 4, 6, 8, 10 μg/mL).

To 5 mL of the test solution, 20.0 mL of acetonitrile-water solution (V:V/84:16) was added. After shaking for 20 min and centrifugation (8,000 rpm, 8 min), 4 mL of the supernatant was taken and subjected to three consecutive extractions with an equal volume of chloroform. The extract was evaporated under nitrogen at 55°C. The precipitate was dissolved in 1 mL of DMSO, filtered through a 0.22 μm organic membrane, and the filtrate was analyzed for AFB_1_ content using High-Performance Liquid Chromatography (HPLC) system (Waters Acquity, Milford, MA, United States).

HPLC conditions for AFB_1_ detection: Equipped with a BEH C18 chromatographic column (100 mm × 2.1 mm, 1.7 μm) and a 360 nm ultraviolet detector. Column temperature: 40°C; mobile phase: acetic acid ammonium/methanol; injection volume: 10 μL; flow rate: 0.2 mL/min.

In both cases, the percentage of AFB_1_ degradation was calculated by following formula, where C was the AFB_1_ Paek area in treatment, and F was the AFB_1_ peak area in control:


(1) $${AFB}_{1}\;\mathrm{degradation}\;\left(\%\right)=\left(1-\frac{C}{F}\right)\times 100\%$$

### 2.3 Removing AFB_1_ from real food samples

The maximum value of AFB_1_ contamination in food was 10 μg/g, we artificially contaminated real food samples to reach this concentration. Experimental samples applied easily contaminated foods like maize, cheese, and peanuts. To prepare these artificially contaminated samples, 10 mL of AFB_1_ standard solution (1,000 μg/mL) was added separately to 1 kg of maize, cheese, and peanuts. Thorough mixing ensured a final AFB_1_ concentration of 10 μg/g. Following this, each 1 kg artificially contaminated food sample received a uniform spray of 100 mL bacterial suspension (10^8^ CFU/mL), followed by shading incubation (37°C, 72 h). Treatment groups comprised M-YUAD7, C-YUAD7, and P-YUAD7. Positive controls included food samples artificially contaminated with AFB_1_ but without the inoculated strain (C-M, C-C, C-P). Negative controls consisted of naturally incubated food samples (N-M, N-C, N-P), and strains were inoculated into food samples without artificial AFB_1_ contamination (UnM-YUAD7, UnC-YUAD7, UnP-YUAD7). All control groups were also incubated without light (37°C, 72 h). The pretreatment steps for detecting AFB_1_ content in actual food samples were as follows: 20 g of the sample was mixed with 180 mL distilled water in a juice extractor, blended at high speed for 30 s, and subsequently filtered through four layers of gauze. The obtained pulverized extract will be retained for subsequent AFB_1_ content detection, following the same procedures as in step 2.2.

### 2.4 Morphological, physiological and biochemical characterization of target strains

For physiological and biochemical identification of the target strain, a conventional microbial biochemical identification kit (Beijing Luqiao, Beijing, China) was employed, following the guidelines of “Bergey’s Manual of Systematic Bacteriology” (Garrity, 1994).

### 2.5 Genome sequencing and annotations

The genomic DNA of YUAD7 was extracted via a SanPrep DNA purification kit (Sangon Biotech, Shanghai, China), following the manufacturer’s guidelines. Subsequently, a combination of PacBio RS II Single Molecule Real Time (SMRT, Pacific Biosciences, MenloPark, CA, United States) and Illumina sequencing platforms (Illumina Novaseq 6000, Shanghai, China) were employed for sequencing. The accession number PRJNA964696 at the US National Center for Biotechnology Information (NCBI) was assigned to the sequence data of *B. amyloliquefaciens* YUAD7.

The CDSs were predicted with gene annotation performed using GO and KEGG (Fang et al., 2020) using sequence alignment tools such as BLAST,^1^ Diamond (Version 0.8.35) and HMMER.^2^ All data were quantified and visualized on the CGView data platform^3^ and the Chiplot online platform.^4^

### 2.6 Isolating components from the target strains

The AFB_1_ degradation capabilities of cell-free supernatant, intracellular extracts, and dead-cell bacterial suspensions were evaluated using a previously described method (Rao et al., 2017; Cai et al., 2020). The cell-free supernatant, intracellular extracts, and bacterial suspension of dead cells were incubated with TSB-AFB_1_ medium (AFB_1_ concentration of 10 μg/mL) at 37°C with 180 rpm shaking for 72 h. Control cultures consisted of TSB medium or sterile phosphate buffer supplemented (PBS) with AFB_1_(concentration of 10 μg/mL). The residual AFB_1_ was then analyzed as previously described.

### 2.7 Degradation of AFB_1_ active components in target strains treated with different treatments

The degradation of AFB_1_ active components in target strains was fractionated into four components, and the effects of proteinase K (PK, 1 mg/mL), sodium dodecyl sulfate (SDS, 1%), PK + SDS, and heat treatment (boiled for 20 min) on the degradation of AFB_1_ were investigated. Subsequently, each component was incubated with sterile PBS containing AFB_1_ at a 10 μg/mL concentration at 37°C. A sterile PBS with AFB_1_ concentration at 10 μg/mL was used as a control. After 72 h, residual AFB_1_ was detected, as described earlier.

### 2.8 Assessment of the toxicity of degradation products of AFB_1_ by target bacterial strains

The survival monitoring assay utilized L-02 standard cells obtained from the Cell Preservation Bank at the Chinese Academy of Sciences in Shanghai, representing a human normal liver cell line. The NC group was cultured in RPMI 1640 medium, while the CC group was cultivated in AFB_1_-RPMI 1640 solution (AFB_1_ concentration at 10 μg/mL). The EG group was exposed to RPMI 1640 medium with 10% degradation solution. Then, L-02 cells were seeded at a density of 100,000 cells per well in a 24-well plate and incubated in RPMI 1640 medium at 37°C for 24 h under a 5% CO_2_ atmosphere to synchronize the population. Subsequently, the medium was replaced with fresh medium containing the test mentioned above samples. The survival monitoring assay was performed using the Vi-cell system (Thermo Scientific, Waltham, MA), and the cell morphology of each sample was compared with that of its corresponding control.

### 2.9 Ames mutagenicity assay

To assess the mutagenic potential of the degradation products, the Ames test using the Genotoxic Ames kit (Iphase Bio Technology, Suzhou, China) following the manufacturer’s instructions and the protocol outlined by Chen et al. (2022). The degradation products obtained from a 72-h co-culture with the AFB_1_-degrading bacterium and AFB_1_ were incubated with *Salmonella Typhimurium* TA100 or TA102 at 37°C for 48 h (EG). The count of *S. typhimurium* colonies was documented, and the results were expressed as the number of revertant colony-forming units (CFUs). Positive controls consisted of samples extracted from TSB medium with AFB_1_(CC), while negative controls included extracts from TSB medium without AFB_1_(NC).

### 2.10 The degradation product extraction and isolation

The degradation liquid was concentrated to afford a crude residue which was suspended in H_2_O and then extracted with petroleum ether, EtOAc, and n-BuOH to afford fractions.

The petroleum ether fraction (50 g) subjected to silica gel column chromatography (200–300 mesh, Qingdao Haiyang Chemical Factory, Qingdao, China) with DCM and MeOH (1:0~0:1, v/v) gradient elution was distributed as two fractions (Fr1, 15 g, Fr2 6.3 g). Fr1(15 g) was chromatographed on an ODS with MeOH/H_2_O (0:1~0:1, v/v), and then separated by preparative HPLC (MeOH/H_2_O, 40:60, V/V; flow rate, 4 mL/min) to yield compounds 1 (tR = 8.7 min, 20.4 mg), 2 (tR = 6.8 min, 14.6 mg).

Fr2(6.3 g) was separated by silica gel column chromatography (cc, 70 × 245 mm) with DCM/MeOH (1:0 ~ 0:1, V/V) to provide two subfractions (Fr2-1, 1.3 g; Fr2-2, 0.8 g). Fr2-1(1.3 g) was further separated by preparative HPLC (MeOH/H_2_O, 35:65, V/V; flow rate, 4 mL/min) to yield 3 (tR = 7.6 min, 26.3 mg), yield 4 (tR = 5.4 min, 10.5 mg).

### 2.11 Detection of the chemical composition of degradation products of AFB_1_

The UPLC-Q-Orbitrap included a UPLC system equipped with an autoinjector and quaternary UPLC pump (Waters Acquity, Milford, MA, United States), and quadrupole/electrostatic field orbitrap high-resolution mass spectrometry (Q-Orbitrap HRMS; Thermo Scientific, Waltham, MA). The chromatograph was equipped with a BEH C18 column (100 mm × 2.1 mm, 1.7 μm) and operated at a column temperature of 40°C. Samples were injected and eluted using a mobile solvent containing mobile phase A, which was an aqueous solution containing 0.1% formic acid and 5 mmol/L ammonium formate, and mobile phase B, a methanol solution containing 0.1% formic acid and 5 mmol/L ammonium formate. The mass spectrometry parameters were set similar to that in previous study (Lai et al., 2023).

### 2.12 Detection of the molecular structure of the degraded product of AFB_1_ and prediction of the AFB_1_ degradation pathway

The ^1^H and ^13^C NMR experiments data were obtained by Bruker DPX-400 spectrometer in dimethyl sulfoxide-*d*6 (DMSO-*d*6; DPX-400, Bruker, Switzerland). The sample was equipped with a 5 mm NORELL NMR tube and operated at 25°C. The data was analyzed using MestreNova software (ver. 14.2, Mestrelab Research, Escondido, CA). Then, BioTransformer 3.0 software^5^ was used to predict the AFB_1_ degradation pathway of YUAD7 based on the structure of degradation products.

### 2.13 Statistical analysis

The results were presented as mean ± standard deviation (SD). Statistical analysis was performed using SPSS software and involved ANOVA followed by Duncan’s test. Different lowercase letters in the bars of each group indicated significant differences between treatments (*p* < 0.05).

## 3 Results

### 3.1 Screening of target strains

As shown in Table 1, 23 strains capable of utilizing coumarin were obtained using coumarin as the sole carbon source for enrichment and acclimation. YUAD7 exhibited the highest degradation rate, achieving a 91.7% degradation within 72 h in TSB-AFB_1_ solution with an AFB_1_ concentration of 10 μg/mL. Therefore, YUAD7 was chosen as the target strain for further research.

**Table 1:** Sources of aflatoxin B_1_ degradation strains and degradation.

No.	Strain no.	Strain source	Degradation of AFB_1_
1	MAAD32	Silage corn in Haixi Mongolian and Tibetan Autonomous Prefecture, Qinghai Province, China	65.72 ± 0.64%
2	ALAD18	Silage alfalfa in Haixi Mongolian and Tibetan Autonomous Prefecture, Qinghai Province, China	73.43 ± 1.03%
3	ALAD20	Silage alfalfa in Haixi Mongolian and Tibetan Autonomous Prefecture, Qinghai Province, China	70.27 ± 1.84%
4	YUAD7	Yak manure from Haixi Mongolian and Tibetan Autonomous Prefecture, Qinghai Province, China	91.70 ± 1.32%
5	YUAD11	Yak manure from Haixi Mongolian and Tibetan Autonomous Prefecture, Qinghai Province, China	87.73 ± 2.11%
6	MAXN23	Silage corn in Xining City, Qinghai Province, China	73.65 ± 1.21%
7	ALXN12	Silage alfalfa in Xining City, Qinghai Province, China	45.21 ± 2.63%
8	YUXN30	Yak manure in Xining City, Qinghai Province, China	86.28 ± 1.58%
9	MAWT8	Silage corn in Tianzhu Tibetan Autonomous County, Wuwei City, Gansu Province, China	79.61 ± 1.31%
10	MAWT14	Silage corn in Tianzhu Tibetan Autonomous County, Wuwei City, Gansu Province, China	69.27 ± 2.75%
11	ALWT17	Silage alfalfa in Tianzhu Tibetan Autonomous County, Wuwei City, Gansu Province, China	83.11 ± 1.27%
12	YUWT23	Yak manure in Tianzhu Tibetan Autonomous County, Wuwei City, Gansu Province, China	53.28 ± 2.09%
13	MADB12	Silage corn in Diebu County, Gannan Prefecture, Gansu Province, China	68.12 ± 0.89%
14	ALDB16	Silage alfalfa in Diebu County, Gannan Prefecture, Gansu Province, China	70.21 ± 1.56%
15	YUDB22	Yak manure in Diebu County, Gannan Prefecture, Gansu Province, China	64.29 ± 2.56%
16	MAXH3	Silage corn in Xiahe County, Gannan Prefecture, Gansu Province, China	77.93 ± 0.89%
17	ALXH27	Silage alfalfa in Xiahe County, Gannan Prefecture, Gansu Province, China	74.94 ± 1.68%
18	YUXH11	Yak manure in Xiahe County, Gannan Prefecture, Gansu Province, China	86.26 ± 3.01%
19	ALGT15	Silage alfalfa in Gaotai County, Zhangye City, Gansu Province, China	73.28 ± 1.48%
20	YUGT38	Yak manure in Gaotai County, Zhangye City, Gansu Province, China	88.32 ± 3.17%
21	MASN	Silage Corn in Sunan County, Zhangye City, Gansu Province, China	46.11 ± 1.55%
22	ALSN	Silage alfalfa in Sunan County, Zhangye City, Gansu Province, China	65.29 ± 1.62%
23	YUSN	Yak manure in Sunan County, Zhangye City, Gansu Province, China	75.48 ± 2.73%

**Figure 1:**
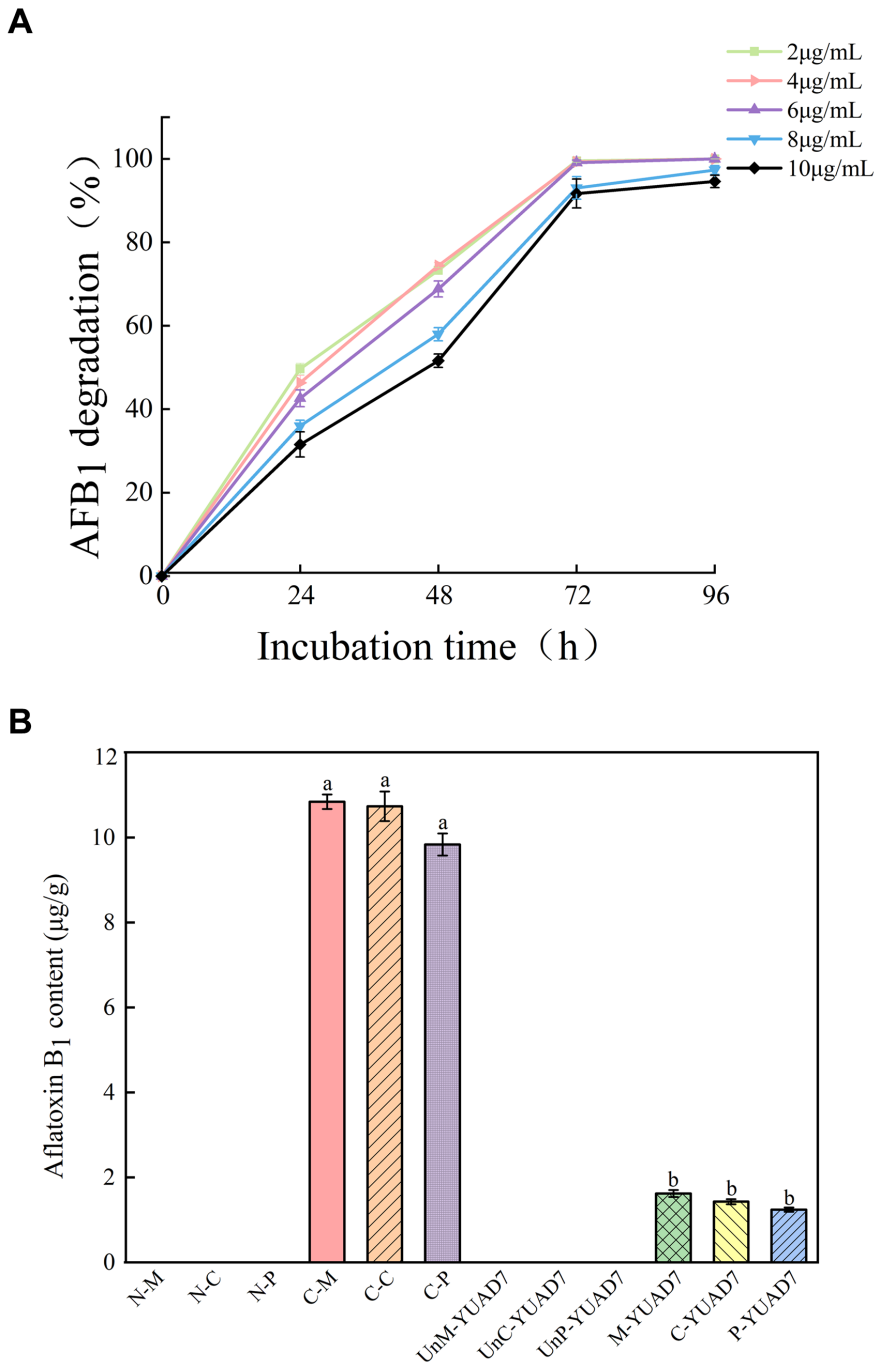
The strain YUAD7 degrades AFB_1_ in different substrates. **(A)** The degradation of different concentrations of AFB_1_ by strain YUAD7 in TSB-AFB_1_ medium. **(B)** YUAD7 degrades AFB_1_ in real food samples.

YUAD7’s ability to degrade AFB_1_ at different concentrations was assessed under consistent incubation time and temperature condition. As depicted in Figure 1A, When the AFB_1_ concentration was below 6 μg/mL, apart from the initial 24-h, the degradation percentages of AFB_1_ by YUAD7 were consistently similar across treatments during the 96-h incubation. The degradation rates nearly reached their maximum at 72-h, exceeding 99%. Furthermore, when the AFB_1_ concentrations were 8 and 10 μg/mL, YUAD7 exhibited time-dependent degradation, achieving a reduction of over 94% by 96-h (AFB_1_ 10 μg/mL). Considering the maximum level in raw cereal grains (Campos-Avelar et al., 2021), 10 μg/mL AFB_1_ was chosen for the subsequent research.

YUAD7 was employed to degrade AFB_1_ in real food samples, as shown in Figure 1B. AFB_1_ levels in all negative control treatments were below the limit of detection [LOD: 0.003 μg/g (mL)]. In the positive controls of maize, cheese, and peanuts, AFB_1_ levels were 10.84 ± 0.17, 10.73 ± 0.35, and 9.83 ± 0.26 μg/g, respectively. After inoculating YUAD7 for AFB_1_ degradation, the levels in maize, cheese, and peanuts were reduced to 1.62 ± 0.08, 1.43 ± 0.06, and 1.24 ± 0.05 μg/g, respectively. When AFB_1_ contamination in food reached 10 μg/g, YUAD7 effectively removed over 85% of AFB_1_ from real food samples within 72 h.

### 3.2 Physiological and biochemical characteristics of the YUAD7 strain

Strain YUAD7 morphological, characteristics physiological and biochemical characteristics were shown in Supplementary Figure 1 and Supplementary Table 1. Referencing Bergey’s Manual of Systematic Bacteriology (Garrity, 1994), the strain YUAD7 was preliminarily identified as belonging to the *Bacillus*.

### 3.3 Genetic characterization of the YUAD7 strain

**Figure 2:**
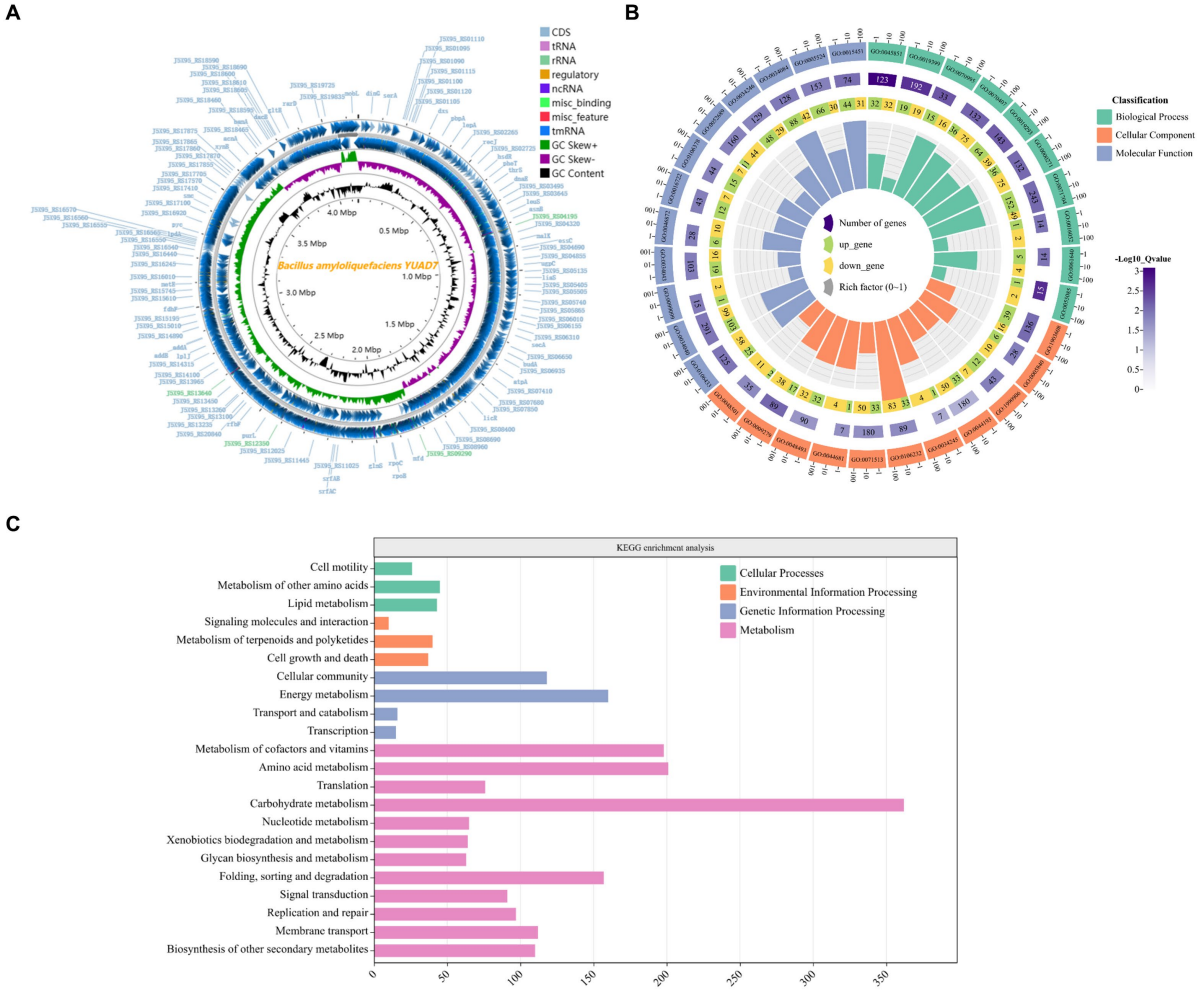
Genome characterization of the YUAD7 strain. **(A)** Circular representation of the *Bacillus amyloliquefaciens* YUAD7 complete genome. From the outmost: 1, Genome structure positive and negative strands; 2, GC Skew; 3, GC content. **(B)** GO analysis of annotated coding genes in *B. amyloliquefaciens* YUAD7 genome. From the outmost: 1, GO ID. 2, Background genes. 3, Upregulated and downregulated genes. 4, Enrichment coefficients. **(C)** KEGG pathway classification of annotated coding genes in *B. amyloliquefaciens* YUAD7 genome.

Whole genome sequencing was performed on strain YUAD7, and the results were shown in Supplementary Table 2 and Figure 2A. The complete genome of YUAD7 was a circular chromosome with a size of 4,028,188 bp and a G + C content of 46.4%. The DNA coding sequences encompassed 3,628,166 bp, constituting approximately 90.07% of the total genome. Following genome assembly and prediction annotation, a total of 4,172 predicted genes were identified, of which 4,054 were CDSs and 118 were RNA genes. Upon manual prediction and annotation, 3,218 and 2,106 predicted genes were annotated in the GO and KEGG databases. Based on the analysis of 16S rRNA and 30 housekeeping genes, including *acnA*, *dacB*, *licR*, *mfd*, *lplJ*, *lpdA*, *lepA*, *secA*, *budA*, *atpA*, *gltB*, *bamA*, *rfbF*, *rpoC*, *rpoB*, *leuS*, *dnaE*, *thrS*, *pheT*, *hsdR*, *recJ*, *pbpA*, *SerA*, *dinG*, *mobl*, *rarD*, *gltB*, *xynB*, *pyc*, and *lpdA*, the closest relative of YUAD7 was *Bacillus*. *amyloliquefaciens* strain (Supplementary Figure 2). Consequently, the strain mentioned above was designated as *Bacillus. amyloliquefaciens* YUAD7.

YUAD7’s CDSs annotation information in the GO and KEGG databases was shown in Figures 2B,C, respectively. Figure 2B indicated that the annotation information of YUAD7’s encoded genes in the GO database mainly fell into three categories: biological process, cellular component, and molecular function, such as oxidation-dependent protein catabolic process (GO: 0070407), polysaccharide biosynthetic process (GO: 0000271), carbohydrate catabolic process (GO: 0044193), hydroxyisourate hydrolase complex (GO: 0106232), O6-methyl-dGTP hydrolase activity (GO: 0106433), and stearyl deacetylase activity (GO: 0034084). Furthermore, in Figure 2C, KEGG annotation information demonstrated that the functions annotated by gene sequences in the primary metabolic pathways could be divided into four categories: cellular processes, environmental information processing, genetic information processing, and metabolism. These findings provide an understanding of the functional genes and metabolic pathways involved in AFB_1_ degradation by *B. amyloliquefaciens* YUAD7 at the genomic level. These predicted metabolic pathways and functional genes were closely associated with the process of AFB_1_ degradation by the strain.

All the data indicated that *B. amyloliquefaciens* YUAD7 could serve as a beneficial and safe bacterium to be applied in food and feed processing.

### 3.4 The active component for AFB_1_ degradation in *Bacillus amyloliquefaciens* YUAD7 and its characteristics

**Figure 3:**
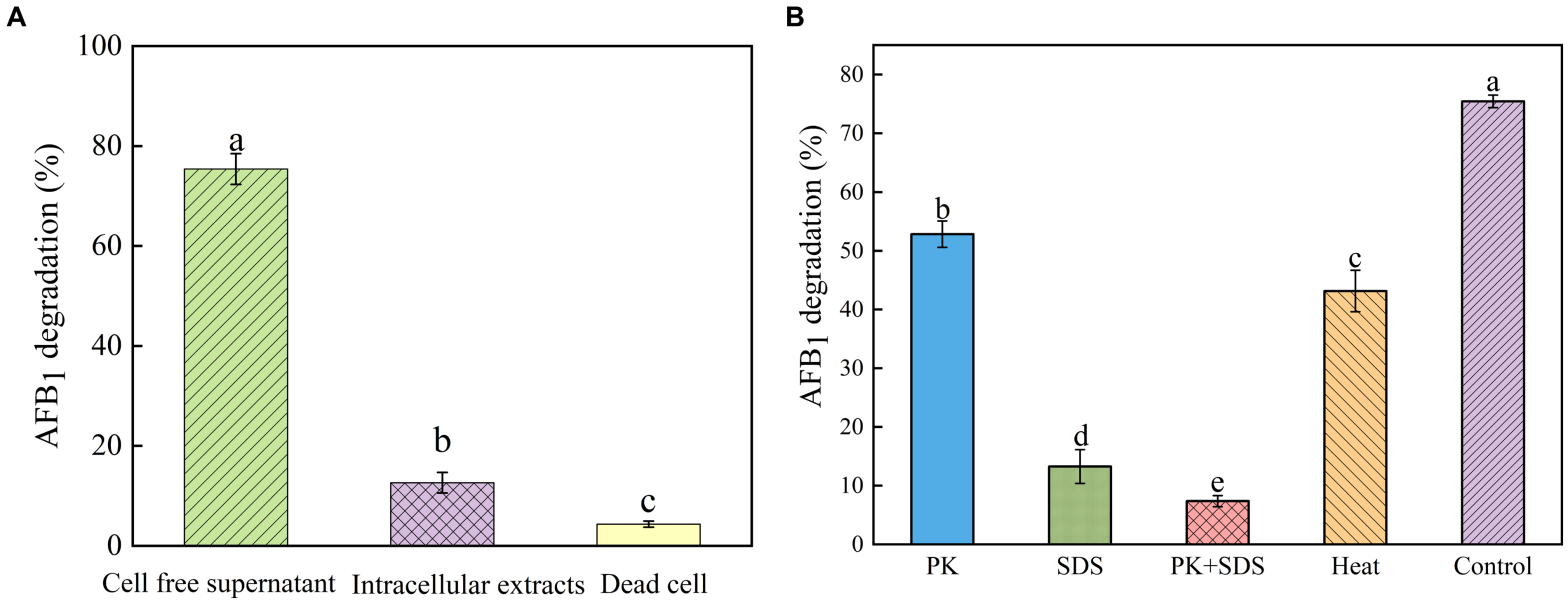
Identification of active substances and factors influencing the activity of strain YUAD7 in the degradation of AFB_1_. **(A)** AFB_1_ degradation by diverse cell components of *Bacillus amyloliquefaciens* YUAD7 during 72-h incubation with 10 μg/mL AFB_1_ at 37°C. **(B)** Effects of heat, PK, SDS, and PK + SDS on AFB_1_ degradation mediated by the cell-free supernatant after co-incubation for 72-h with 10 μg/mL AFB_1_ at 37°C.

In order to investigate the mechanism of AFB_1_ removal by *B. amyloliquefaciens* YUAD7, the study tested the efficiency of cell-free supernatant, intracellular extracts, and dead cells in degrading AFB_1_. As shown in Figure 3A, after 72-h of cultivation, cell-free supernatant could remove 75.42% ± 3.09% of AFB_1_ (10 μg/mL), while the removal rates for dead cells and intracellular extracts were 4.36% ± 0.61% and 12.63% ± 2.05%, respectively. The cell-free supernatant of *B. amyloliquefaciens* YUAD7 was more effective in reducing AFB_1_ than dead cells and intracellular extracts (*p* < 0.05). These results suggested that the removal of AFB_1_ by *B. amyloliquefaciens* YUAD7 was mainly dependent on degradation, and the cell-free supernatant was the main active component in the AFB_1_ degradation process. SDS, proteinase K (PK), and PK + SDS effected on the activity of YUAD7’s cell-free supernatant in degrading AFB_1_ was shown in Figure 3B, AFB_1_ degradation capacity of the cell-free supernatant rated down to 52.85% ± 2.25%, 13.26% ± 2.88%, and 7.36% ± 0.95%. The result indicated that the AFB_1_ degradation agents in *B. amyloliquefaciens* YUAD7 cell-free supernatant included not only enzymes or proteins but also other non-protein components. Additionally, the cell-free supernatant still exhibited AFB_1_ degradation activity of 43.16 ± 3.54% even after boiling for 20 min (Figure 3B).

### 3.5 Toxicity of the products of AFB_1_ degradation by strain YUAD7

**Figure 4:**
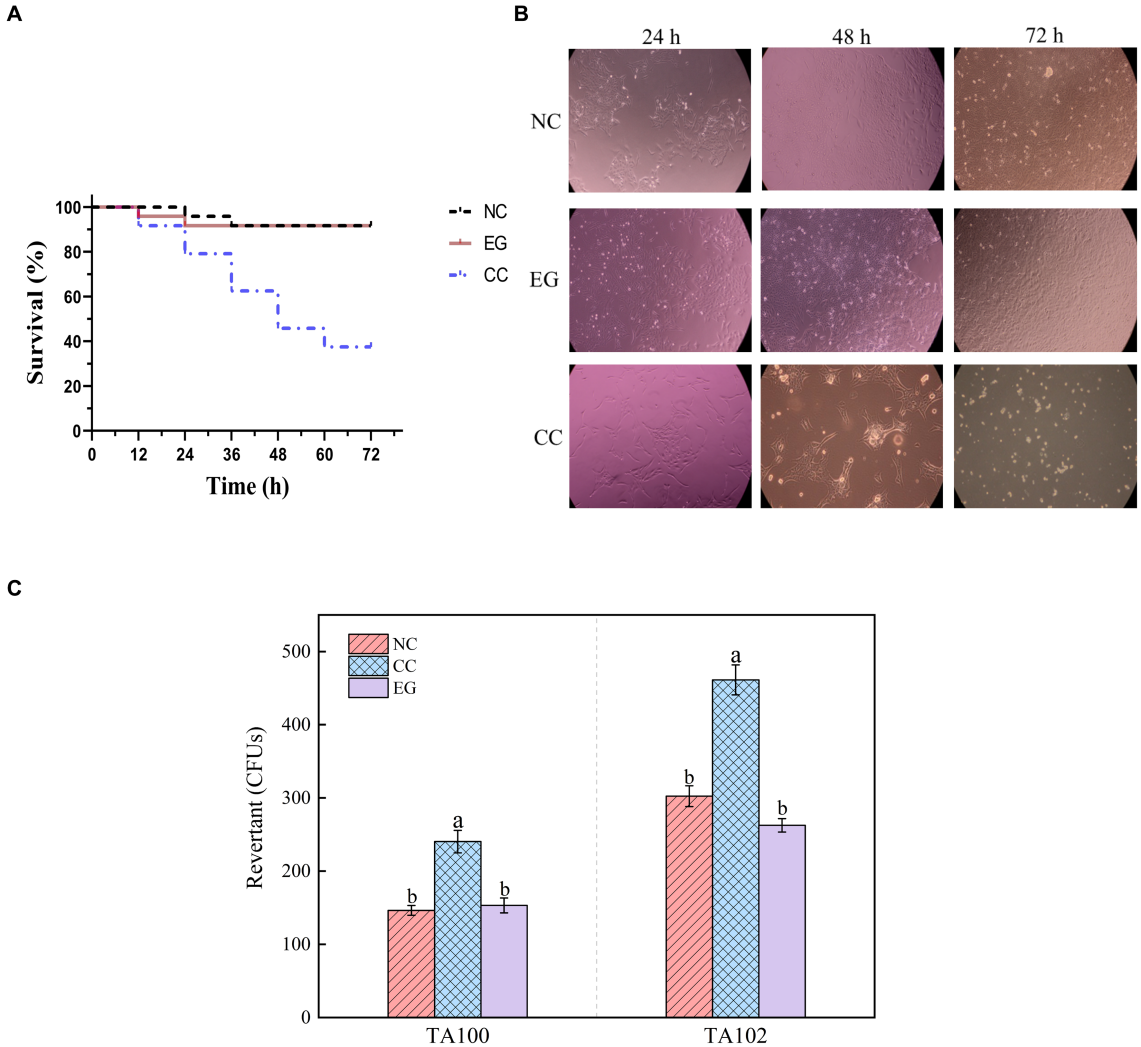
Toxicological results of YUAD7 degradation products. **(A)** Changes in the lifespan of L-02 cells caused by different treatment groups. **(B)** Changes in cell morphology in different treatment groups within 72 h. **(C)** The Ames mutagenicity assay. NC group: medium without AFB_1_. EG group: medium with AFB_1_ degradation liquid of YUAD7. CC group: medium with AFB_1_.

The study examined the impact of AFB_1_ and its degradation products by *B. amyloliquefaciens* YUAD7 on the lifespan of L-02 cells. As shown in Figure 4A, there was no statistical difference (*p* > 0.05) in cell viability between the EG group and the NC group, with L-02 cell survival rates exceeding 92%. However, the CC group exhibited a 62.5% decrease in the average lifespan of L-02 cells. The morphological changes of L-02 cells under different treatment conditions within 72-h of cultivation were shown in Figure 4B. Compared to the NC group, the EG group showed no significant differences in cell morphology (*p* > 0.05). In contrast, the CC group exhibited cell elongation, lysis phenomena, and a significant reduction in cell viability. These results showed that *B. amyloliquefaciens* YUAD7 degraded AFB_1_ into metabolites without toxicity to the L-02 cells. Meanwhile, the Ames test was employed to evaluate the mutagenic potential of AFB_1_ degradation products facilitated by *B. amyloliquefaciens* YUAD7. Compared to the control group, a roughly twofold increase in the count of revertant CFUs from *S. typhimurium* TA100 and TA102 was noted in the AFB_1_ group (CC). However, there was no statistically significant difference in revertant CFUs between the degradation products (EG) and the control group (NC; Figure 4C), indicating that *B. amyloliquefaciens YUAD7* transformed AFB_1_ into metabolites with diminished mutagenicity.

### 3.6 Identification and analysis of AFB_1_ metabolic degradation products

**Figure 5:**
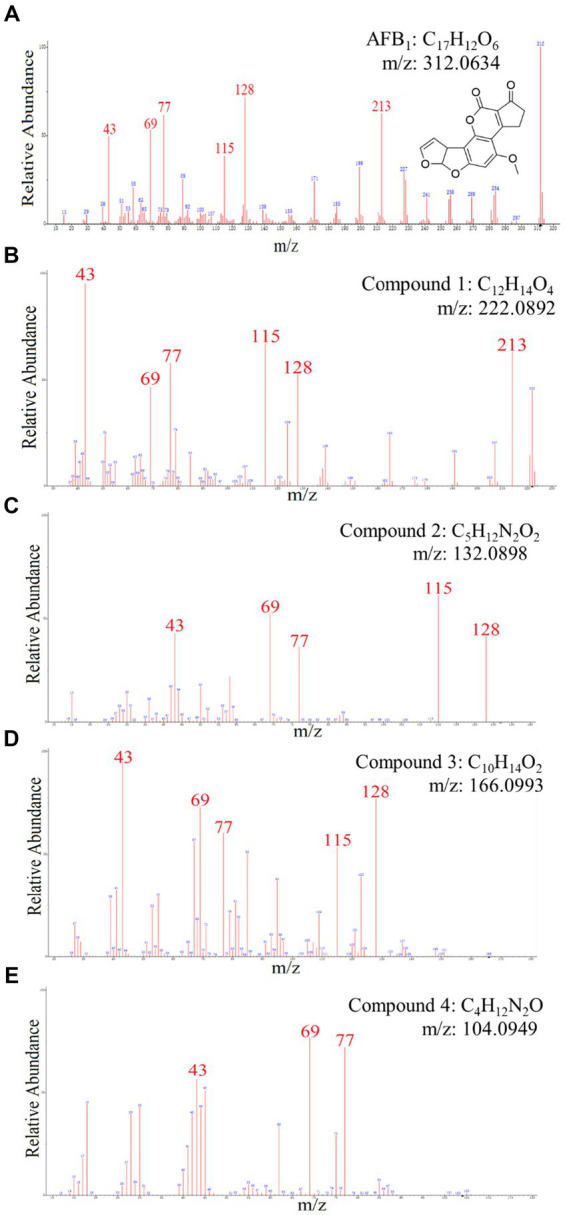
The mass spectra and chemical formula of AFB_1_ standards and the compounds in degradation solution. **(A)** C_17_H_12_O_6_. **(B)** C_12_H_14_O_4_. **(C)** C_5_H_12_N_2_O_2_. **(D)** C_10_H_14_O_2_. **(E)** C_4_H_12_N_2_O.

Chemical components of AFB_1_ degradation products by strain YUAD7 were analyzed using the UPLC-Q-Orbitrap HRMS method. The characteristic ions of AFB_1_ standard compounds, which were m/z 213, 128, 115, 77, 69, and 43, respectively (Figure 5A). Comparing the fragment ions of AFB_1_ and the compounds in degradation solution, compound 1–4 had high homology with the fragment ions of AFB_1_, and compound 1–4 were the products of the degradation of AFB_1_ by the *B. amyloliquefaciens* YUAD7 (Supplementary Figure 3).

**Figure 6:**
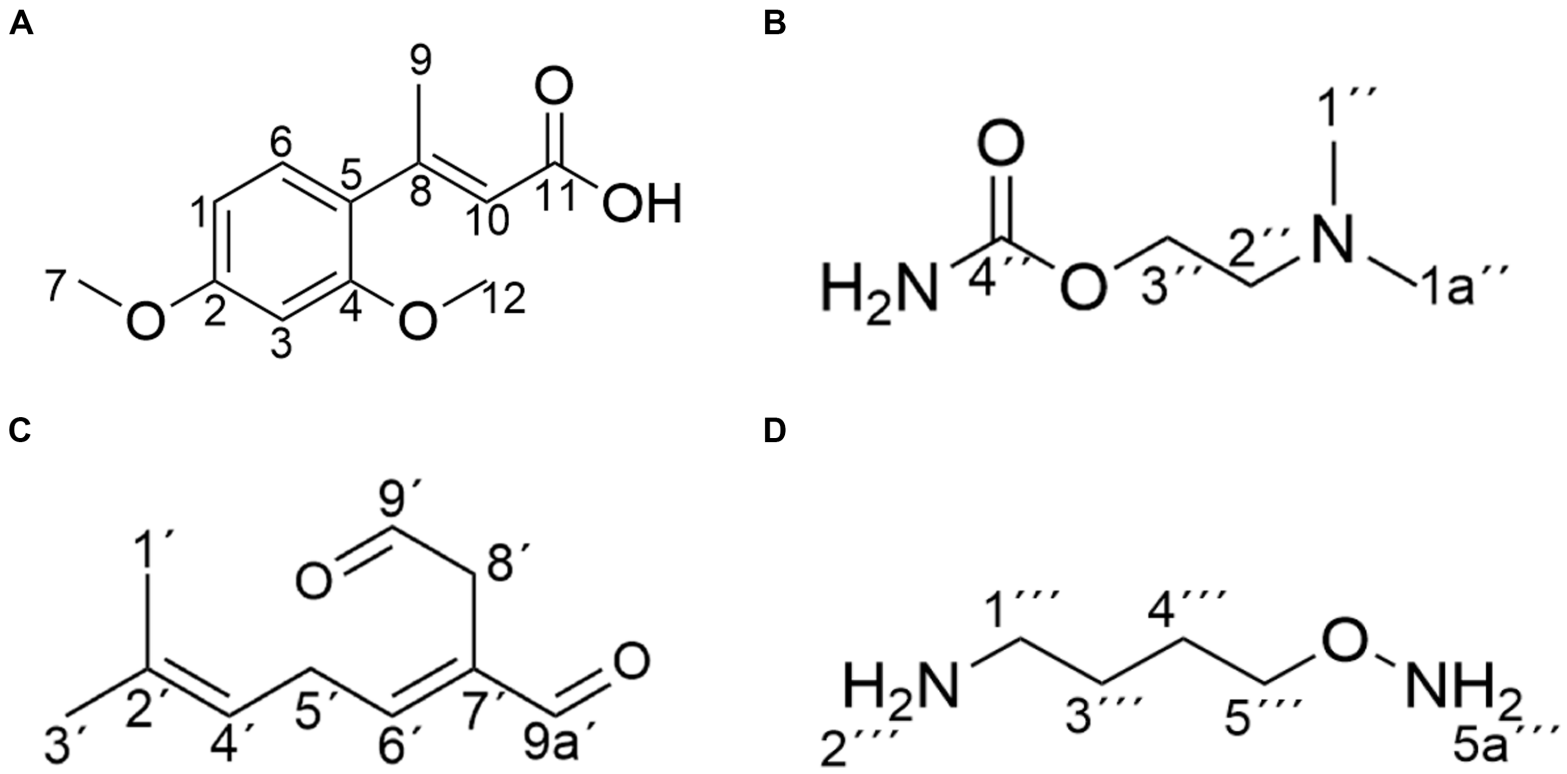
Structure of the main products of AFB_1_ degradation by strain YUAD7. **(A)** C_12_H_14_O_4_. **(B)** C_5_H_12_N_2_O_2_. **(C)** C_10_H_14_O_2_. **(D)** C_4_H_12_N_2_O.

Compound 1 was isolated as white powder. Its molecular formula was established as C_12_H_14_O_4_ by UPLC-Q-Qrbitrap HRMS (m/z 222.0892), sharing several homologous fragment ions with AFB_1_, such as 213, 128, 115, 77, 69, 43, etc. (Figure 5B). Its ^1^H NMR spectrum (Table 2) showed characteristic signals for the hydrogen of ABX coupling system on the benzene ring [δ_H_7.34 (1H, d, J = 8.2 Hz, H-6), 6.75(1H, dd, J = 8.1, 2.3 Hz, H-1), and 6.52(1H, d, J = 2.2 Hz, H-3)]; δ_H_3.80(3H, s, H-7), 2.42(3H, s, H-9),3.86(3H, s, H-12)] were three methoxy hydrogen signals. δ_H_6.14 (1H, q, J = 1.3 Hz, H-10) was the hypomethyl hydrogen signal. Analysis of the ^13^C NMR data (Table 2) revealed 12 carbon signals, including 6 carbon signals on the benzene ring, three methoxy carbon signals (δ_C_55.67, 20.44, 55.92), and three sp^3^ hybridized carbon data[δ_C_153.39(C-8), 115.33(C-10), 170.01(C-11)] which indicated that compound 1 had the basic skeleton of dimethoxy benzene. The determination of the -CH linkage position and its sequential arrangement within compound 1 was determined by HSQC spectra (Supplementary Figure 6). The benzene ring structure suggested by HSQC correlations from δ_H_7.34 (1H, d, J = 8.2 Hz, H-6) to δ_C_129.36(C-6), δ_H_6.75(1H, dd, J = 8.1, 2.3 Hz, H-1) to δ_C_107.87(C-1), δ_H_6.52(1H, d, J = 2.2 Hz, H-3) to δ_C_97.85(C-3). The three methyl groups were at C7, C9 and C12 positions, respectively, [δ_H_3.80 (3H, s) to δ_C_55.67(C-7), δ_H_2.42 (3H, s) to δ_C_20.44(C-9), δ_H_3.86 (3H, s) to δ_C_55.92(C-12). Finally, the hydrocarbon formation pertaining to compound 1 was precisely ascribed through the chemical formula, ^1^H NMR, ^13^C NMR, HSQC, and the structure of compound 1 was (2-4-dimethoxyphenyl) but-2-enoic acid (Figure 6A).

**Table 2:** ^1^H NMR (500 MHz) and ^13^C NMR (125 MHz) Data of 1–4 in DMSO-*d*6 (δ in ppm, J in Hz).

No	1		2		3		4	
	δ_H_	δ_C_	δ_H_	δ_C_	δ_H_	δ_C_	δ_H_	δ_C_
1	6.75 (dd, J = 8.1, 2.3, 1H)	107.87						
2		161.64						
3	6.52 (d, J = 2.2, 1H)	97.85						
4		160.68						
5		120.64						
6	7.34 (d, J = 8.2, 1H)	129.36						
7	3.80 (s, 3H)	55.67						
8		153.39						
9	2.42 (s, 3H)	20.44						
10	6.14 (q, J = 1.3, 1H)	115.33						
11		170.01						
12	3.86 (s, 3H)	55.92						
1″			2.38 (s, 3H)	45.89				
1a″			2.38 (s, 3H)	45.89				
2″			2.89 (t, J = 6.7, 2H)	58.52				
3″			4.26 (t, J = 6.7, 2H)	62.73				
4″			4.88 (s, 2H)	157.14				
1′					1.67–1.61 (m, 3H)	18.03		
2′						132.41		
3′					1.67–1.61 (m, 3H)	25.59		
4′					5.38 (th, J = 6.6, 1.6, 1H)	122.49		
5′					2.74 (dddq, J = 7.1, 6.2, 2.0, 1.0, 2H)	27.09		
6′					6.51 (tq, J = 5.9, 1.4, 1H)	143.36		
7′						137.16		
8′					3.00 (dq, J = 5.8, 1.0, 2H)	40.40		
9′					9.83–9.75 (m, 1H)	200.36		
9a′					9.83–9.75 (m, 1H)	194.32		
1‴							2.88–2.68 (m, 2H)	41.58
2‴							1.43 (t, J = 6.5, 2H)	
3‴							1.82–1.51 (m, 2H)	29.71
4‴							1.82–1.51 (m, 2H)	25.19
5‴							3.53 (d, J = 11.7, 2H)	73.17
5a‴							5.45 (s, 2H)	

Compound 2 was isolated as a yellow powder with the molecular formula C_5_H_12_N_2_O_2_, which was established by UPLC-Q-Orbitrap HRMS (m/z 132.0898), sharing several homologous fragment ions with AFB_1_ such as 128, 115, 77, 69, 43, etc. (Figure 5C). The ^1^H NMR data (Table 2) of 2 showed the hydrogen signal of two methoxies [δ_H_ 2.38 (3H, s, H-1″, H-1a´´)]. δ_H_ 2.89 (2H, t, J = 6.7 Hz, H-2″) and 4.26 (2H, t, J = 6.7 Hz, H-3″) were two hydrogen signals on methylene, and δ_H_ 4.88 (2H, s, H-4″) was the nitrile hydrogen signal. ^13^C NMR spectra (Table 2) showed the presence of 5 carbon signals. Among them, 2 signals belonged to 2 methoxies [δ_C_ 45.89 (C-1″) and (C-1a″)]. δ_C_ 58.52 (C-2″) and 62.73(C-3″) were the methylene carbon signal. δ_C_157.14 (C-4″) were the aminoester group carbon signal. Based on the above date, as well as HSQC data (Supplementary Figure 9), 2 was speculated 2-(dimethylamino) ethyl carbamate (Figure 6B).

Compound 3 was obtained as a yellow powder and the UPLC-Q-Orbitrap HRMS data of 3 (m/z 166.0993, calcd for C_10_H_14_O_2_) indicated its molecular formula of C_10_H_14_O_2_, sharing several homologous fragment ions with AFB_1_ such as 128, 115, 77, 69, 43, etc. (Figure 5D). In the ^1^H NMR data (Table 2) δ_H_ 1.67–1.61(3H, m, H-1′, H-3′) were the characteristic hydrogen signals of the methoxy. δ_H_ 5.38 (1H, th, J = 6.6, 1.6 Hz, H-4′) and 6.51(1H, tq, J = 5.9,1.4 Hz, H-6′) were two methenyl hydrogen signals. δ_H_ 2.74 (2H, dddq, J = 7.1, 6.2, 2.0, 1.0 Hz, H-5′) and 3.00 (2H, dp, J = 5.8,1.0 Hz, H-8′) were two hydrogen signals on methylene. δ_H_ 9.83–9.75 (1H, m, H-9′, H-9a′) were two formyl hydrogen signals. The ^13^C NMR data (Table 2) of 3 showed 10 carbon signals, including 2 methoxy hydrogen signals, 2 methene signals, 2 methenyl signals, 2 formyl signals, and 2 other sp3 hybrid carbon signals. The above HRMS and NMR data (Table 2; Supplementary Figure 12) indicated that 3 was polyunsaturated fatty acid structure (Figure 6C).

Compound 4 was isolated as a yellow powder and its molecular formula was deduced as C_4_H_12_N_2_O on the basis of its UPLC-Q-Orbitrap HRMS data (m/z 104.0949), sharing several homologous fragment ions with AFB_1_ such as 77, 69, 43 etc. (Figure 5E). The ^1^H NMR data (Table 2; Supplementary Figure 15) of 4 showed the hydrogen signals of the aminomethyl group [δ_H_ 2.88–2.68 (2H, m, H-1‴), 1.43 (2H, t, J = 6.5 Hz, H-2‴)]. δ_H_ 1.82–1.51(2H, m, H-3‴, H-4‴) and δ_H_ 3.53 (2H, d, J = 11.7, H-5‴) were the hydrogen signals of methylen. δ_H_ 5.45 (2H, s, H-5a‴) was the aminogruppe hydrogen signal. The ^13^C NMR data (Table 2) of 4 showed 4 carbon signals, including 3 carbon signals on methylene signals, 1 aminomethyl group signal. The compound 4 was the 1-aminooxy-4-aminobutane (Figure 6D).

The structure of degradation products by the strain were determined using NMR as shown in Figure 6. The chemical structures of the products were primarily consisted of dimethoxyphenyl and enoic acid (compound 1), dimethylamino and ethyl carbamate (compound 2), polyunsaturated fatty acid (compound 3), and aminomethyl (compound 4). Among these four product structures, there were no structures that were associated with the high toxicity of AFB_1_, including the furan ring double bond, coumarin lactone ring, and cyclopentenone ring.

### 3.7 Prediction of AFB_1_ degradation pathway by YUAD7 strain

**Figure 7:**
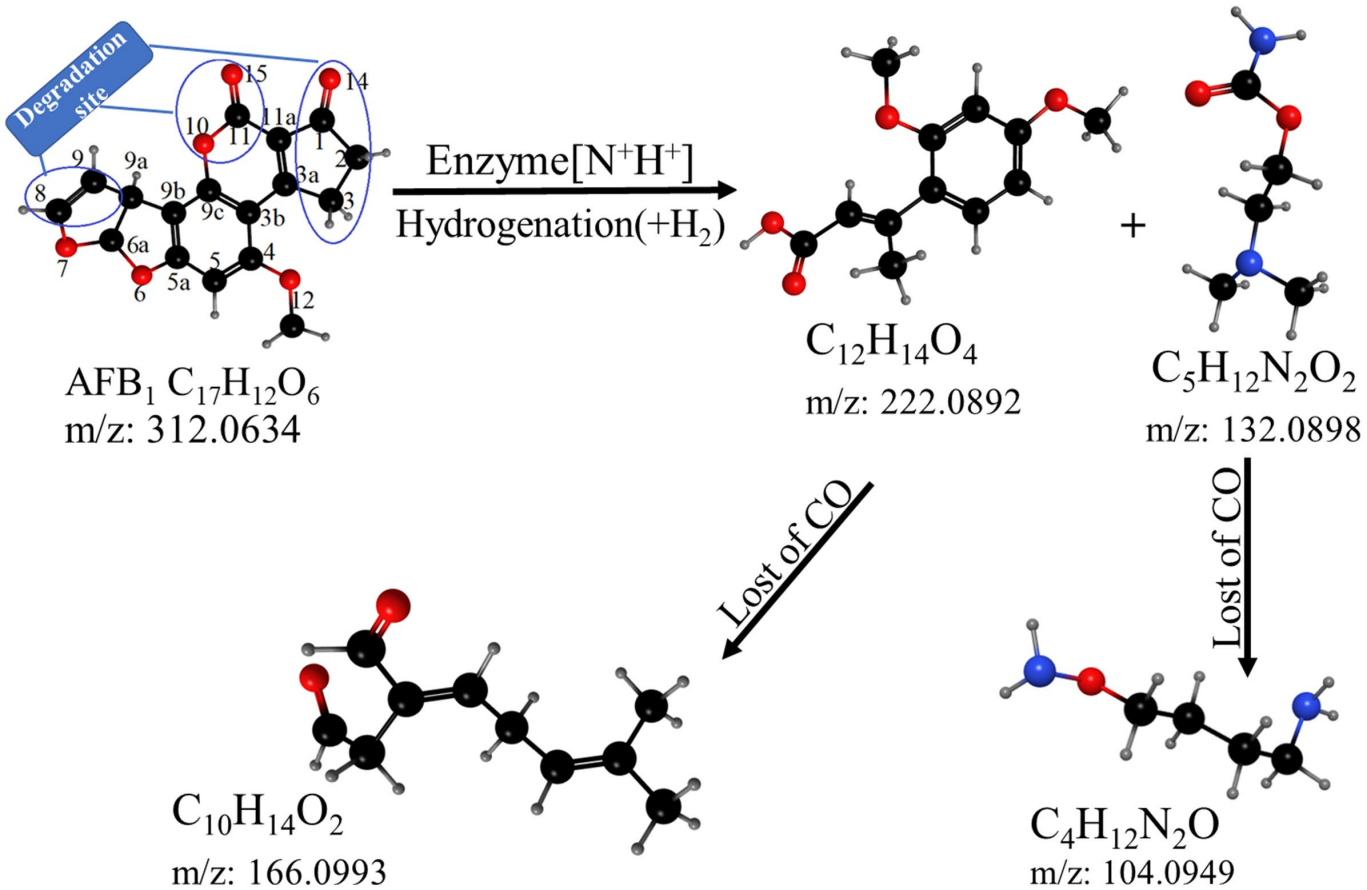
The hypothetical pathway of AFB_1_ degradation by YUAD7.

Based on the structure of the degradation products, it could be inferred that *B. amyloliquefaciens* YUAD7 mainly degraded AFB_1_ through secondary degradation (Figure 7). Primary degradation was achieved through hydrogenation and enzyme modification, directly cleaving the coumarin moiety (at positions 10, 11, and 15) and the cyclopentenone ring (at position 14) structures from the AFB_1_ parent structure. Simultaneously, the modification disrupted the furan ring structure at positions 8 and 9, resulting in the formation of compound C_12_H_14_O_4_. Moreover, an enzymatic modification added reactions that collided with the [N^+^H^+^] ion precursor bound, possibly forming the compound C_5_H_12_N_2_O_2_. Secondary degradation involved further decomposition of the primary degradation products C_12_H_14_O_4_ and C_5_H_12_N_2_O_2_, primarily through the removal of the -CO moiety. In this process, the products underwent additional structural adjustments and cleavage, forming simpler compounds such as C_10_H_14_O_2_ and C_4_H_12_N_2_O.

## 4 Discussion

The *B. amyloliquefaciens* YUAD7 strains, isolated from Tibetan Plateau yak manure in this study, exhibited remarkable degradation capabilities of AFB_1_. *Bacillus amyloliquefaciens* YUAD7 could degrade AFB_1_ at concentrations ranging from 1 to 6 μg/mL by more than 99% and 8 to 10 μg/mL by more than 91% after a 72-h incubation. These results surpassed the 84% reduction of AFB_1_ concentration at 1–5 μg/mL reported for *B. amyloliquefaciens* WF2020 (Chen et al., 2022) and were notably higher than the 73 and 40% reductions reported for *B. amyloliquefaciens* B10 (Xiong et al., 2022) and S8C (Ali et al., 2021), respectively. Moreover, after 96 h of incubation, YUAD7 exhibited an impressive degradation of 94.60% for AFB_1_ (10 μg/mL). Among the *Bacillus* Spp. with the highest reported degradation efficiency, including *B. amyloliquefaciens* B10 (Xiong et al., 2022), *B. amyloliquefaciens* WF2020 (Chen et al., 2022), *B. velezensis* AD8 (Zhao et al., 2022), *B. albus* YUN5 (Kumar et al., 2023), *B. subtilis* JSW-1 (Xia et al., 2017), and *B. licheniformis* CFR1 (Rao et al., 2017), none achieved efficiency exceeding 90% in previous studies. The utilization of *B. amyloliquefaciens* YUAD7 held the promise of significant time and cost reductions, making this process more effective. In terms of ensuring the safety of probiotic microorganisms in food and feed, the FAO/WHO guidelines emphasized that probiotics were the best microorganisms to include in food and feed (Song et al., 2022). *Bacillus amyloliquefaciens* was recognized as an intestinal probiotic for humans and mammals (Marchese et al., 2018).

*Bacillus amyloliquefaciens* YUAD7 primarily removed AFB_1_ through degradation, with extracellular proteins or enzymes as the main active substances. This finding consisted with previous research on AFB_1_ degradation by Bacillus species, including *B. amyloliquefaciens* WF2020 (Chen et al., 2022), *B. licheniformis* CFR1 (Rao et al., 2017), *B. velezensis* DY3108 (Shu et al., 2018), and *B. mojavensis* RC3B (Gonzalez et al., 2019). Moreover, the cell-free supernatant of *B. amyloliquefaciens* YUAD7 could still degrade AFB_1_ by 43.2% after boiling for 20 min, which was higher than that of *B. amyloliquefaciens* WF2020 (Chen et al., 2022). Since mesophilic bacteria or enzymes often failed to endure the harsh reaction conditions required in industrial processes, it was highly beneficial that thermostable extracellular proteins or enzymes might provide robust and efficient catalyst substitutes that could withstand the harsh reaction conditions required in industrial processes.

Previous studies have reported the complete genome sequences of other Bacillus strains (Fang et al., 2020; Chen et al., 2022), however, these analyses needed more comprehensive annotation of functional genes and metabolic pathways. The functional bacteria research had indicated that through whole-genome annotation, the *Enterobacter roggenkampii* ED5 strain predicted metabolic processes and functional genes related to biological control (Guo D. J. et al., 2020). In this study, based on whole-genome prediction, metabolic processes related to AFB_1_ degradation included cellular processes, environmental information processing, genetic information processing, and metabolism. Functional genes involved were the oxidation-dependent protein catabolic process (GO: 0070407), polysaccharide biosynthetic process (GO: 0000271), and carbohydrate catabolic process (GO: 0044193). The full-genome analysis of *B. amyloliquefaciens* YUAD7 represented a valuable tool for identifying and categorizing genes involved in the biodegradation of AFB_1_, enabling a comprehensive insight into the functional genes and metabolic pathways underlying this process.

Meanwhile, the safety of microorganisms in food and feed was ensured through toxicological analysis of YUAD7 degradation products. As the AFB_1_ toxin caused the most damage to liver cells (Alvarez et al., 2019), normal human hepatocytes L-02 were chosen as the experimental subjects for the study to respond more sensitively to the toxicity of the degradation products on cells. The results were similar to those of studies on *B. licheniformis* ANSB821 (Guo Y. et al., 2020), which degraded AFB_1_ products assaying to L-02 cells. In summary, using *B. amyloliquefaciens* YUAD7 for AFB_1_ degradation was safe, as both the strain and the degradation products were non-toxic. Non-toxic products contributed to reducing the processing steps for by-products during the removal of AFB_1_ contamination in food and feed processing and ensured the safety of the degradation process.

The hydrogenation degradation pathway of YUAD7 was similar to the degradation pathway found by *Aspergillus niger* FS10. The *Aspergillus niger* FS10 degraded AFB_1_ by successive hydrolysis-decarboxylation, breaking down the large AFB_1_ molecule into non-toxic small molecules and removing the methoxy group from the benzene ring (Qiu et al., 2021). The enzymatic modification degradation pathway was similar to the degradation of AFB_1_ by *B. licheniformis* ANSB821, in which product presence of K and Na elements in the degradation products could result from enzyme binding to AFB_1_ through modification, addition to AFB_1_ molecules, and formation of [N^+^K^+^] and [N^+^Na^+^] ion precursors through collisions (Guo Y. et al., 2020). *Candida versatilis* CGMCC 3790 degraded AFB_1_ through the addition and hydrolysis pathway, resulting in four products with a chemical formula similar to that of YUAD7 degradation products (Li et al., 2018). Through structural analysis of metabolites and speculation of metabolic pathways, it confirmed that YUAD7 degradation sites were the double bonds of the furan ring, vanillin endolipid ring, and pentenone ring structure of AFB_1_, and the carcinogenic, teratogenic, and mutagenic toxicity sites in AFB_1_ was degraded through hydrolysis, enzyme modification, and loss of the -CO group for biological detoxification.

In the future, the incorporation of ^14^C labeling technology will be anticipated, enabling the labeling of C atoms in AFB_1_ and subsequently facilitating the meticulous tracking of its intricate degradation trajectory.

## 5 Conclusion

*Bacillus amyloliquefaciens* YUAD7, isolated from the extreme environment of the Qinghai-Tibet Plateau, can efficiently degrade AFB_1_ at 10 μg/mL, with a remarkable 91.7% efficiency within 72 h. It also removes over 85.0% of AFB_1_ from real food samples (AFB_1_ concentration 10 μg/g) within the same timeframe, establishing it as one of the most effective strains for degrading high AFB_1_ concentrations. The YUAD7 strain primarily degraded AFB_1_ through extracellular secretions and exhibited excellent thermal stability. Furthermore, *B. amyloliquefaciens* YUAD7 transformed AFB_1_ into non-toxic small molecular compounds, including C_12_H_14_O_4_, C_5_H_12_N_2_O_2_, C_10_H_14_O_2_, and C_4_H_12_N_2_O, through processes such as hydrogenation, enzyme modification, and the loss of the -CO group. This capability was valuable for reducing AFB_1_ contamination in food and feed processing. However, the degradation pathway of AFB_1_ by the YUAD7 strain was inferred based on the structure of the degradation products in this study. In the future, ^14^C tracing technology will be employed to meticulously trace the degradation pathway of YUAD7 in AFB_1_ within real food samples. This will provide a more precise understanding of the degradation metabolic pathway, offering technical support for the application of YUAD7.

## Data availability statement

The datasets presented in this study can be found in online repositories. The names of the repository/repositories and accession number(s) can be found in the article/Supplementary material.

## Ethics statement

Ethical approval was not required for the studies on animals in accordance with the local legislation and institutional requirements because only commercially available established cell lines were used.

## Author contributions

YT: Conceptualization, Data curation, Investigation, Methodology, Software, Validation, Writing – original draft, Writing – review & editing. XL: Conceptualization, Funding acquisition, Writing – review & editing. LD: Data curation, Methodology, Writing – original draft. SH: Data curation, Methodology, Writing – original draft.

## Footnotes

^1^ https://blast.ncbi.nlm.nih.gov/Blast.cgi

^2^ http://www.hmmer.org

^3^ https://paulstothard.github.io/cgview

^4^ http://www.chiplot.online

^5^ https://biotransformer.ca
