# Supplementary material for: Screening and identification of an aflatoxin B1-degrading strain from the Qinghai-Tibet Plateau and biodegradation products analysis
Source: Front Microbiol. 2024 May 1;15:1367297. doi: 10.3389/fmicb.2024.1367297 (PMC11094616; doi:10.3389/fmicb.2024.1367297)
Supplement: Supplementary file 1 [file Data_Sheet_1.pdf]

Supplementary Material

Screening and Identification of an Aflatoxin B_1-Dergrading Strain from the Qinghai-Tibet Plateau and Biodegradation Products Analysis

Ying Tang[1], Xiaojing Liu[1*], Ling Dong[1] and Shengran He[1]

[1] College of Pratacultural science, Gan Su Agricultural University, Lanzhou, China.

*** Correspondence:**
Xiaojing Liu
liuxj@gsau.edu.cn

Supplementary Figures and Tables

Physiological and biochemical identification **3**

Supplementary Figure 1. Strain YUAD7 Morphological Characteristics. (A) Colonies. (B) Gram stain of bacterial strains. (C) Strain Spore Staining 3

Supplementary Table 1. Physiological and biochemical characteristics of stain YUAD7 3

Phylogenetic tree **4**

Supplementary Figure 2. Phylogenetic tree built using Neighbor-Joining (NJ) method base on the 16S rRNA gene sequence of strain YUAD7 and the sequences of representative strains from GenBank. The bar represents 0.10 substitutions per site. *Paenibacillus macquariensis* ATCC 23464 was used as an outgroup. 4

Genomic characterization **4**

Supplementary Table 2. Genomic characterization and gene annotation of strain YUAD7 4

The total ion chromatograms **5**

Supplementary Figure 3. The total ion chromatograms (TIC) before and after treating the cell-free supernatant co-incubated with TSB-AFB_1 solution 5

1D and 2D NMR spectra of compound 1 .... **6**

Supplementary Figure 4. ^{1}H NMR (500 MHz) spectrum of Compound **1** in DMSO-*d*6 6

Supplementary Figure 5.^{13}C NMR (125 MHz) spectrum of Compound **1** in DMSO-*d*6 6

Supplementary Figure 6. HSQC spectrum of Compound **1** in DMSO-*d*6........ 7

1D and 2D NMR spectra of compound 2 .... **7**

Supplementary Figure 7. ^{1}H NMR (500 MHz) spectrum of Compound **2** in DMSO-*d*6 7

Supplementary Figure 8.^{13}C NMR (125 MHz) spectrum of Compound **2** in DMSO-*d*6 8

Supplementary Figure 9. HSQC spectrum of Compound **2** in DMSO-*d*6........ 8

1D and 2D NMR spectra of compound 3 .... **9**

Supplementary Figure 10. ^{1}H NMR (500 MHz) spectrum of Compound **3** in DMSO-*d*6 9

Supplementary Figure 11.^{13}C NMR (125 MHz) spectrum of Compound **3** in DMSO-*d*6 9

Supplementary Figure 12. HSQC spectrum of Compound **3** in DMSO-*d*6........ 10

1D and 2D NMR spectra of compound 4 .... **10**

Supplementary Figure 13. ^{1}H NMR (500 MHz) spectrum of Compound **4** in DMSO-*d*6 10

Supplementary Figure 14.^{13}C NMR (125 MHz) spectrum of Compound **4** in DMSO-*d*6 11

Supplementary Figure 15. HSQC spectrum of Compound **4** in DMSO-*d*6........ 11

Supplementary Figures and Tables

Physiological and biochemical identification

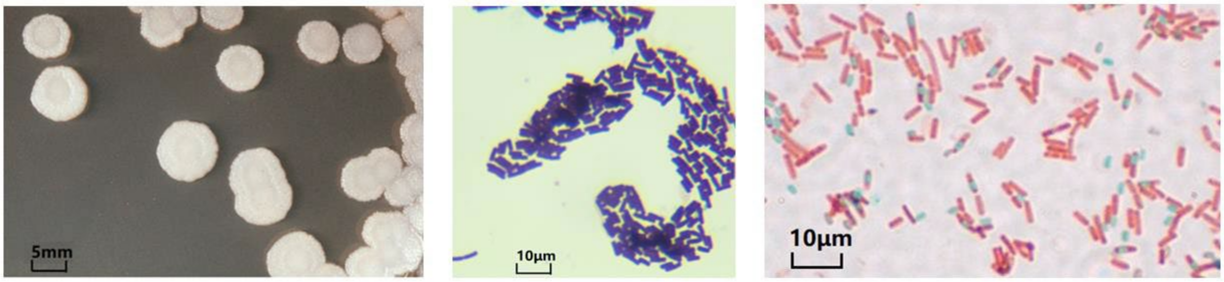

(A) (B) (C)

Supplementary Figure 1. Strain YUAD7 Morphological Characteristics. (A) Colonies. (B) Gram stain of bacterial strains. (C) Strain Spore Staining

Supplementary Table 1. Physiological and biochemical characteristics of stain YUAD7

Item	Result	Item	Result
GLU acid production	+	caprolactam	+
GLU gas production	-	lactose	-
peptone water	-	lysine	+
mannitol	+	ornithine	+
phenylalanine	-	contact enzyme	-
V-P	-	H_2S	-

Phylogenetic tree

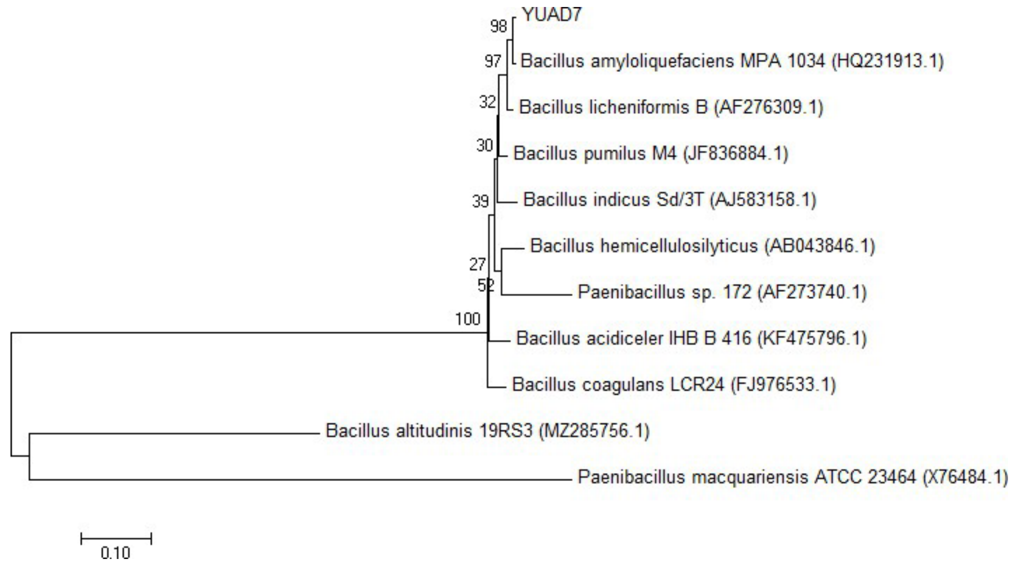

Supplementary Figure 2. Phylogenetic tree built using Neighbor-Joining (NJ) method base on the 16S rRNA gene sequence of strain YUAD7 and the sequences of representative strains from GenBank. The bar represents 0.10 substitutions per site. *Paenibacillus macquariensis* ATCC 23464 was used as an outgroup.

Genomic characterization

Supplementary Table 2. Genomic characterization and gene annotation of strain YUAD7

Characteristic	Value	% of total
Genome size（bp）	4,028,188	100
DNA coding（bp）	3,628,166	90.07
C+G content(bp)	1,869,079	46.40
Genes(total)	4172	100
Coding sequences (CDSs)	4054	97.17
RNA sequences	118	2.83
Topology	Circular	/
Plasmid	0	/
Pseudo Genes(total)	76	1.82
Genes assigned to GO	3218	88.66
Genes assigned to KEGG	2106	51.77
Genes with Biological Process	1041	24.95
Genes with Cellular Component	884	21.19
Genes with Molecular Function	1293	30.99
Genes with Cellular Processes	114	2.73
Genes with Environmental Information Processing	87	2.08
Genes with Genetic Information Processing	309	7.41
Genes with Metabolism	1596	38.26

The total ion chromatograms

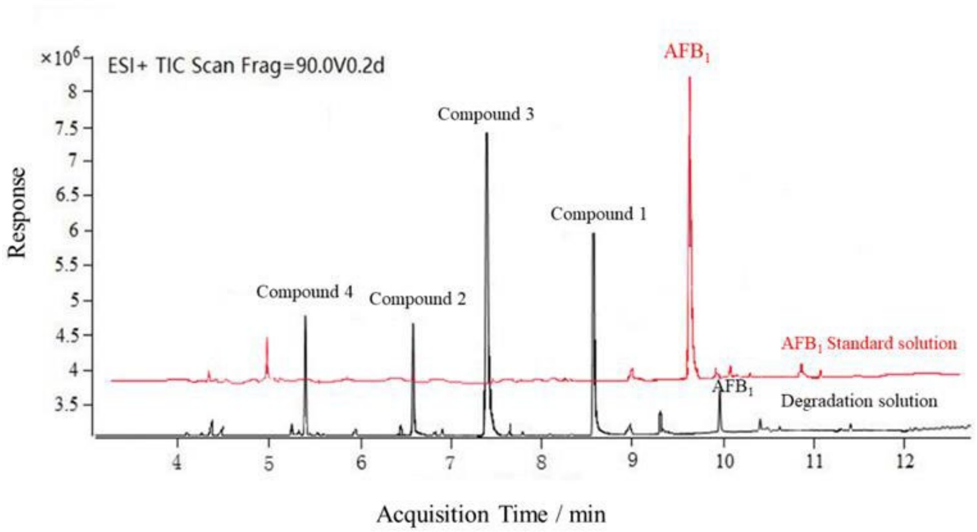

Supplementary Figure 3. The total ion chromatograms (TIC) before and after treating the cell-free supernatant co-incubated with TSB-AFB_1 solution

1D and 2D NMR spectra of compound 1

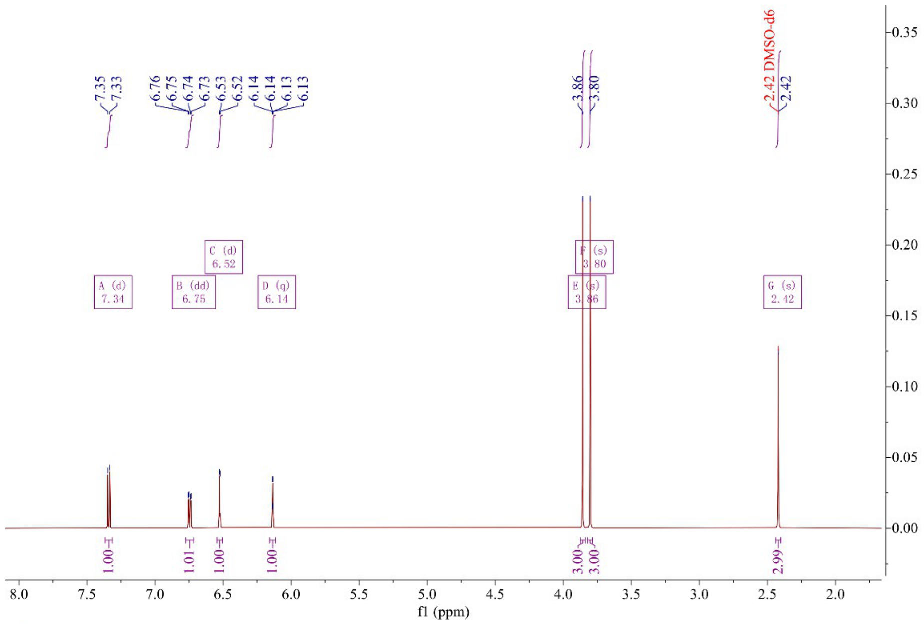

^1H NMR (500 MHz, DMSO-*d6*) δ 7.34 (d, *J* = 8.2 Hz, 1H), 6.75 (dd, *J* = 8.1, 2.3 Hz, 1H), 6.52 (d, *J* = 2.2 Hz, 1H), 6.14 (q, *J* = 1.3 Hz, 1H), 3.86 (s, 3H), 3.80 (s, 3H), 2.42 (s, 3H).

Supplementary Figure 4. ^1H NMR (500 MHz) spectrum of Compound **1** in DMSO-*d*6

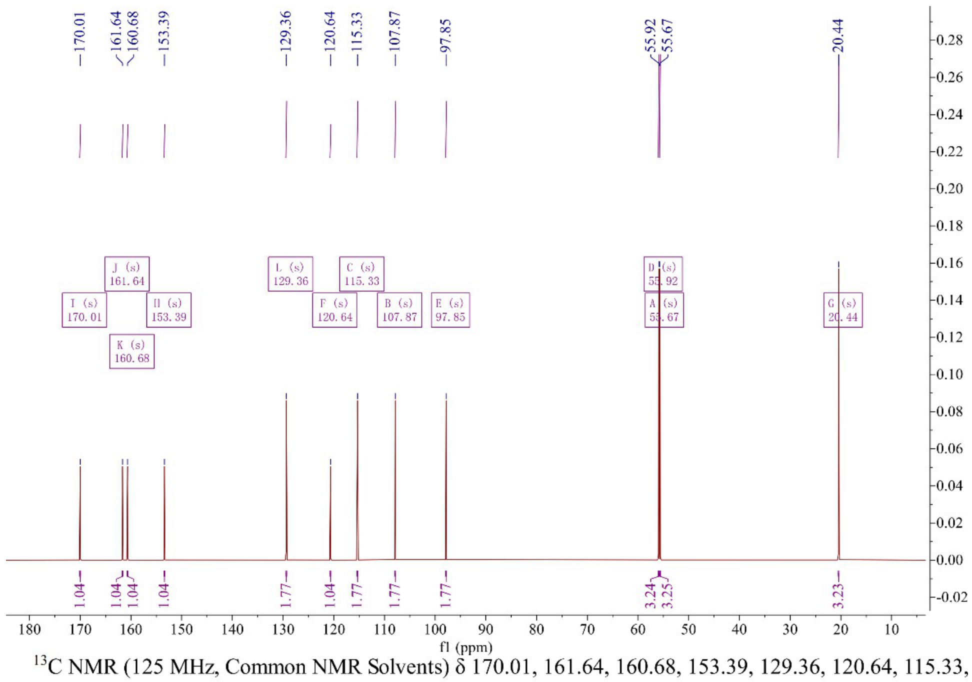

^{13}C NMR (125 MHz, Common NMR Solvents) δ 170.01, 161.64, 160.68, 153.39, 129.36, 120.64, 115.33, 107.87, 97.85, 55.92, 55.67, 20.44.

Supplementary Figure 5. ^{13}C NMR (125 MHz) spectrum of Compound **1** in DMSO-*d*6

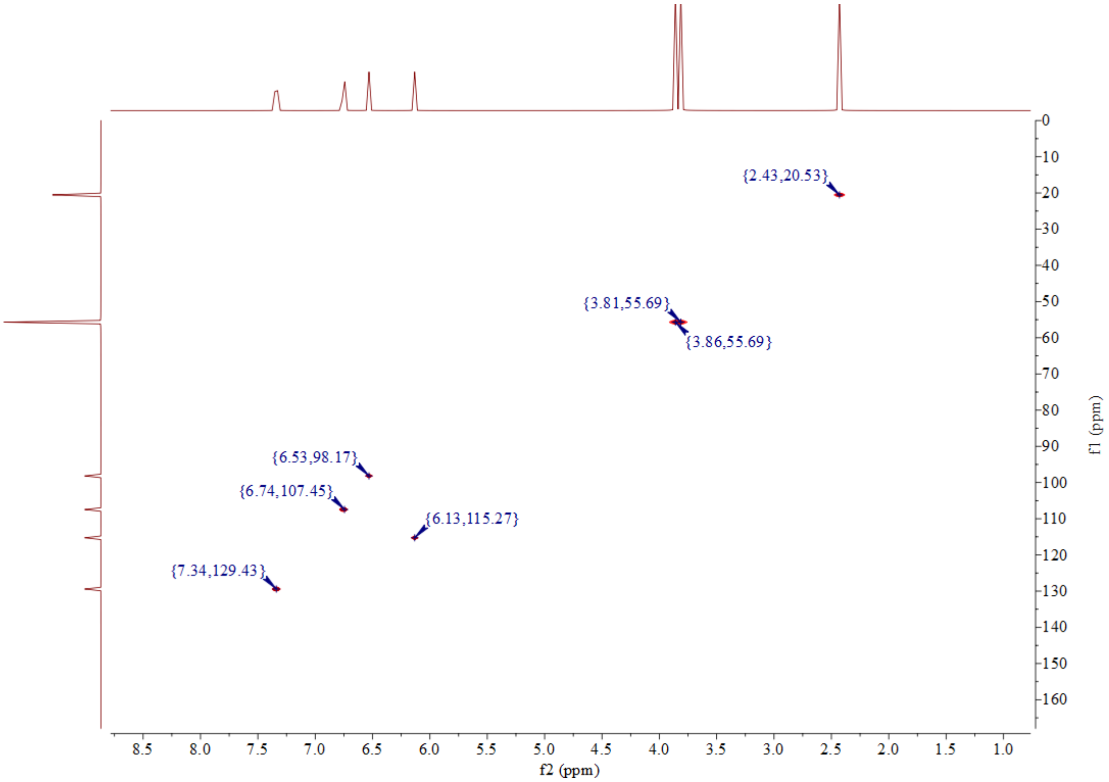

Supplementary Figure 6. HSQC spectrum of Compound **1** in DMSO-*d*6

1D and 2D NMR spectra of compound 2

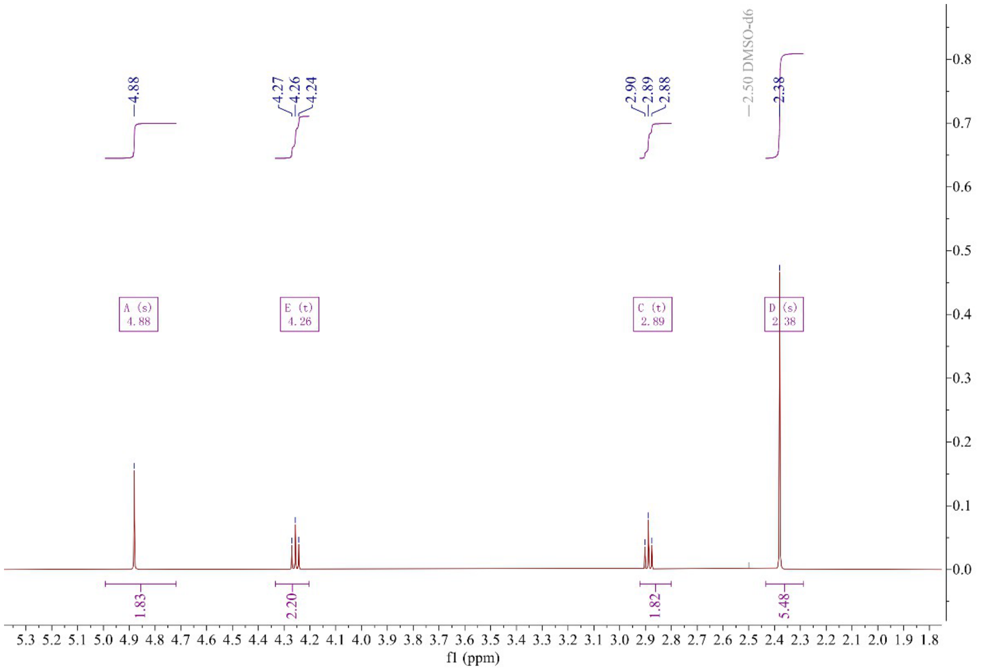

^{1}H NMR (500 MHz, DMSO-*d* 6) δ 4.88 (s, 2H), 4.26 (t, *J* = 6.7 Hz, 2H), 2.89 (t, *J* = 6.7 Hz, 2H), 2.38 (s, 6H).

Supplementary Figure 7. ^{1}H NMR (500 MHz) spectrum of Compound **2** in DMSO-*d*6

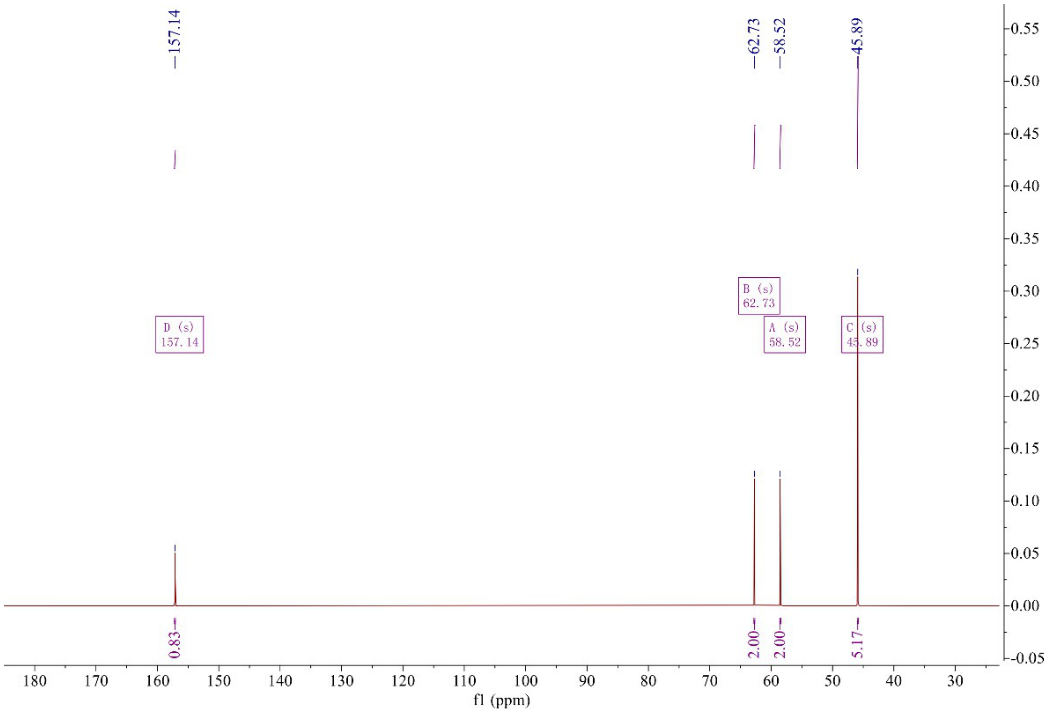

^{13}C NMR (125 MHz, Common NMR Solvents) δ 157.14, 62.73, 58.52, 45.89.

Supplementary Figure 8.^{13}C NMR (125 MHz) spectrum of Compound **2** in DMSO-*d*6

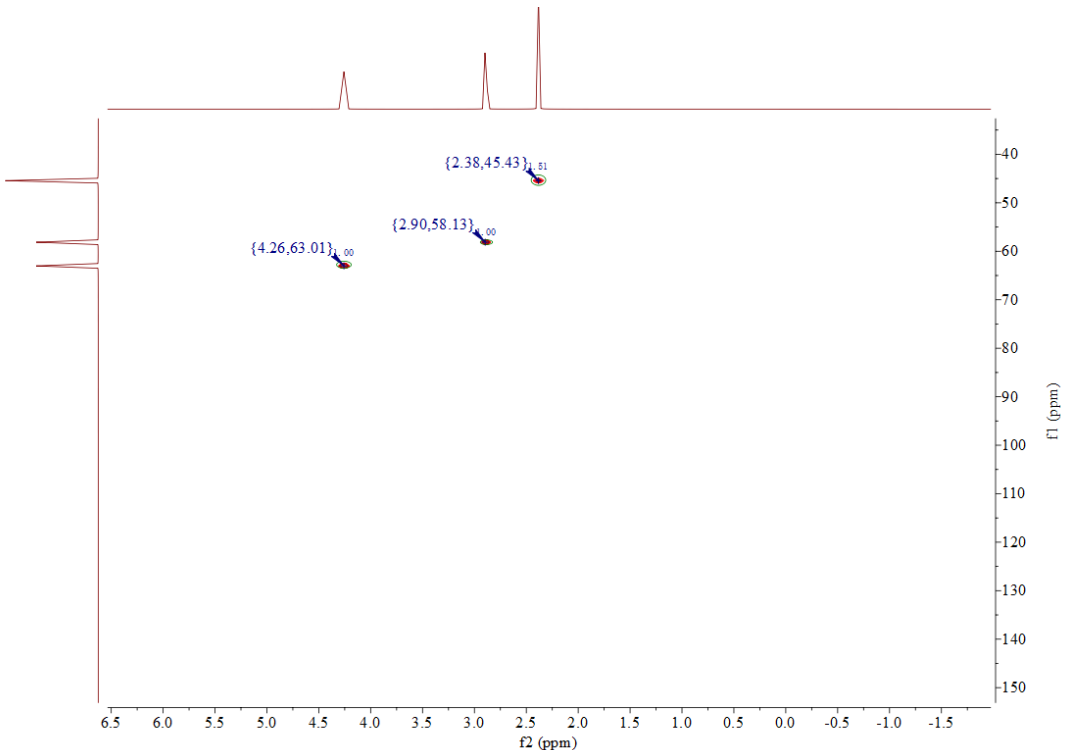

Supplementary Figure 9. HSQC spectrum of Compound **2** in DMSO-*d*6

1D and 2D NMR spectra of compound 3

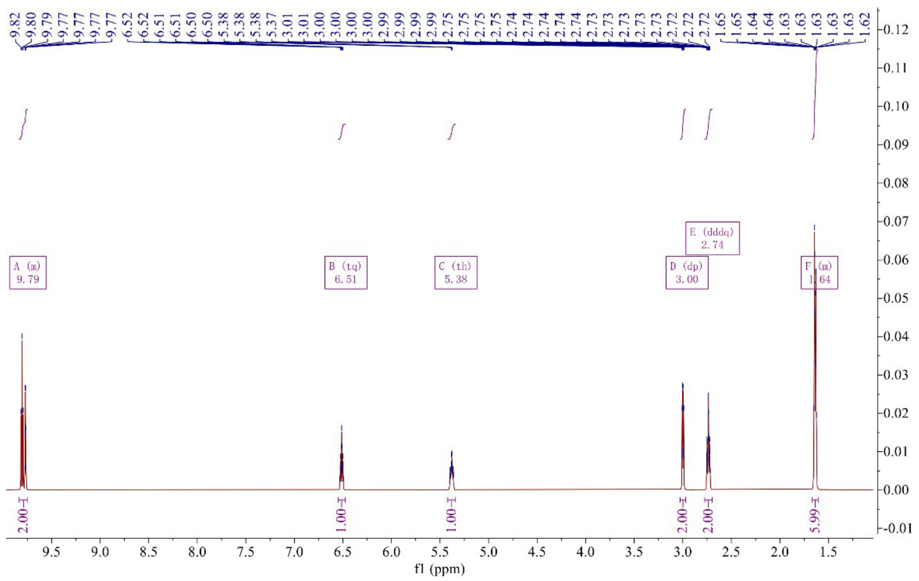

^{1}H NMR (500 MHz, DMSO-*d6*) δ 9.83 – 9.75 (m, 2H), 6.51 (tq, *J* = 5.9, 1.4 Hz, 1H), 5.38 (th, *J* = 6.6, 1.6 Hz, 1H), 3.00 (dp, *J* = 5.8, 1.0 Hz, 2H), 2.74 (dddq, *J* = 7.1, 6.2, 2.0, 1.0 Hz, 2H), 1.67 – 1.61 (m, 6H).

Supplementary Figure 10. ^{1}H NMR (500 MHz) spectrum of Compound **3** in DMSO-*d6*

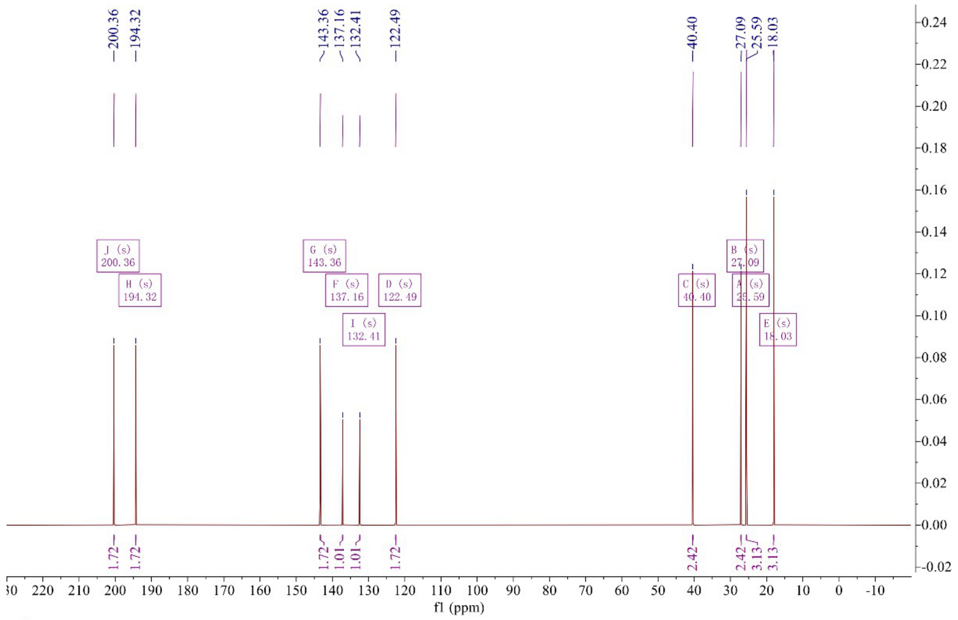

^{13}C NMR (125 MHz, Common NMR Solvents) δ 200.36, 194.32, 143.36, 137.16, 132.41, 122.49, 40.40, 27.09, 25.59, 18.03.

Supplementary Figure 11. ^{13}C NMR (125 MHz) spectrum of Compound **3** in DMSO-*d6*

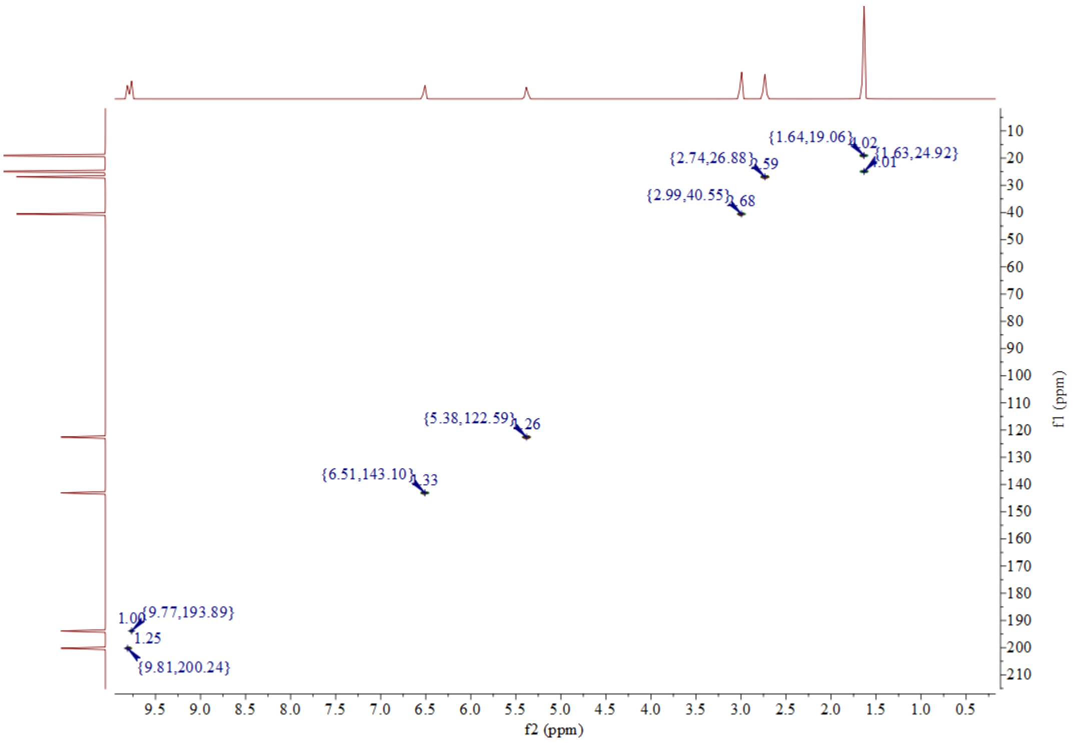

Supplementary Figure 12. HSQC spectrum of Compound **3** in DMSO-*d*6

1D and 2D NMR spectra of compound 4

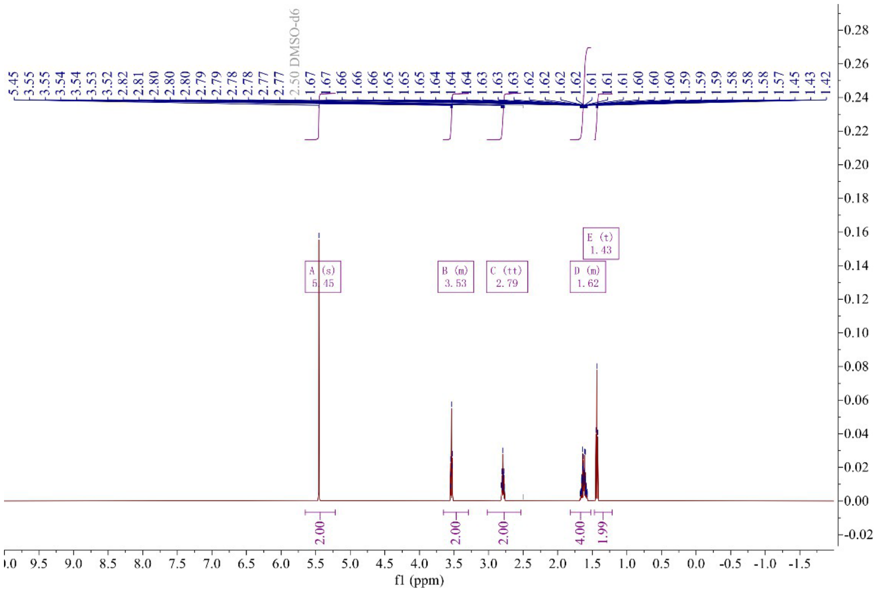

^{1}H NMR (500 MHz, DMSO-*d6*) δ 5.45 (s, 2H), 3.53 (d, J = 11.7 Hz, 2H), 2.88 – 2.68 (m, 2H), 1.82 – 1.51 (m, 4H), 1.43 (t, J = 6.5 Hz, 2H).

Supplementary Figure 13. ^{1}H NMR (500 MHz) spectrum of Compound **4** in DMSO-*d*6

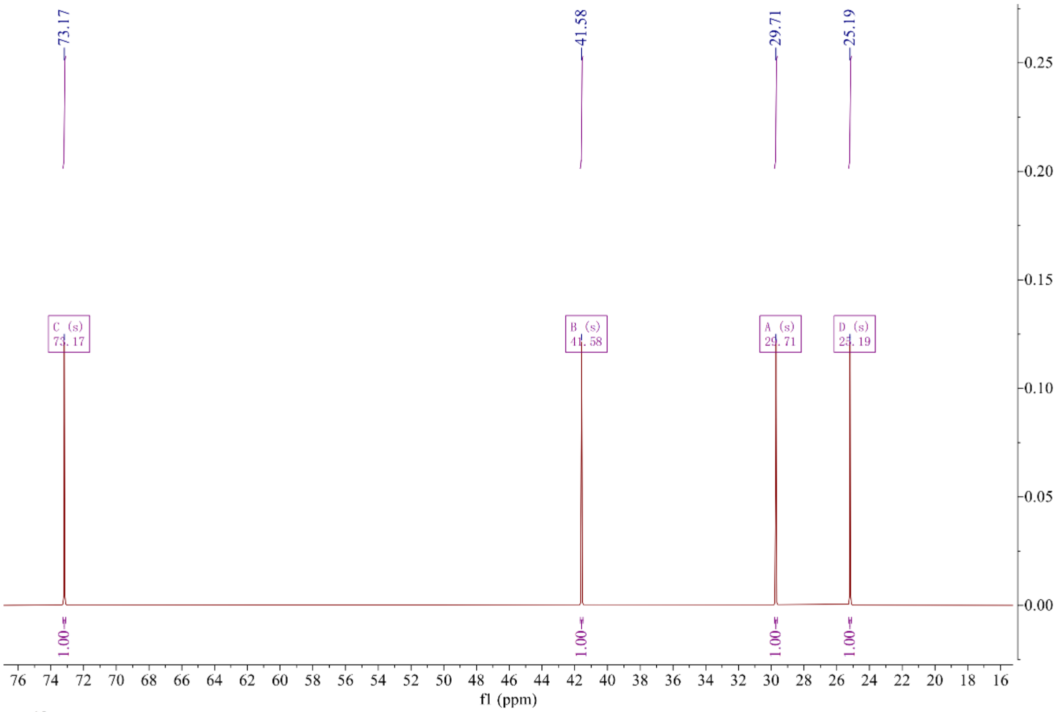

^{13}C NMR (125 MHz, Common NMR Solvents) δ 73.17, 41.58, 29.71, 25.19.

Supplementary Figure 14. ^{13}C NMR (125 MHz) spectrum of Compound **4** in DMSO-*d*6

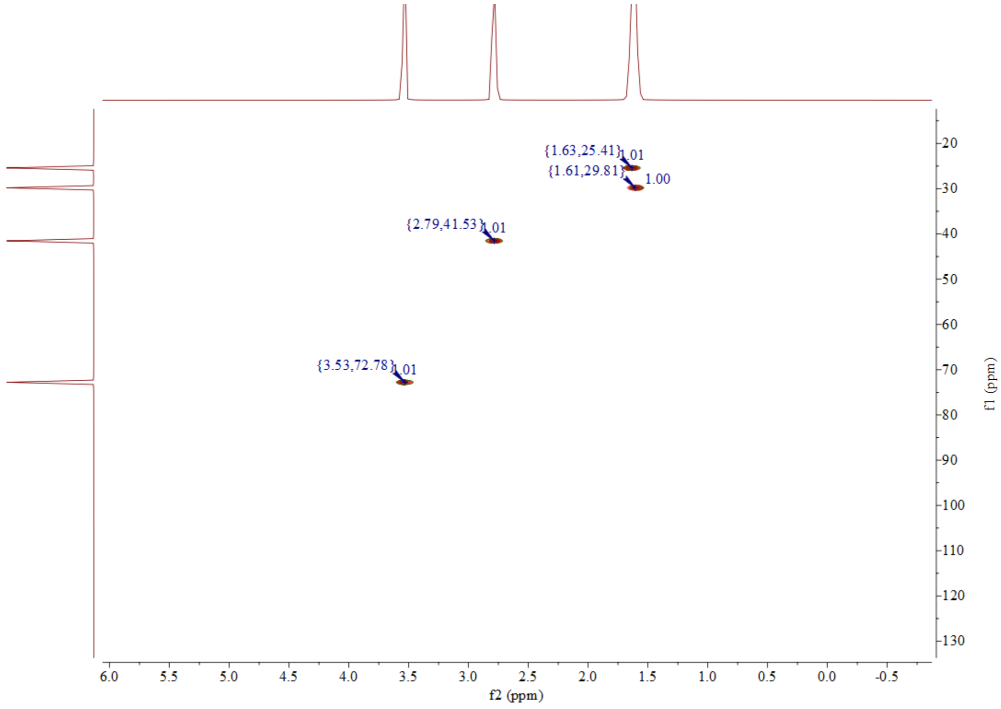

Supplementary Figure 15. HSQC spectrum of Compound **4** in DMSO-*d*6
